# An improved statistical method to identify chemical-genetic interactions by exploiting concentration-dependence

**DOI:** 10.1371/journal.pone.0257911

**Published:** 2021-10-01

**Authors:** Esha Dutta, Michael A. DeJesus, Nadine Ruecker, Anisha Zaveri, Eun-Ik Koh, Christopher M. Sassetti, Dirk Schnappinger, Thomas R. Ioerger

**Affiliations:** 1 Department of Computer Science, Texas A&M University, College Station, TX, United States of America; 2 Laboratory of Host-Pathogen Biology, The Rockefeller University, New York, NY, United States of America; 3 Department of Microbiology and Immunology, Weill Cornell Medical College, New York, NY, United States of America; 4 Department of Microbiology & Physiological Systems, University of Massachusetts Medical School, Worchester, MA, United States of America; Rutgers New Jersey Medical School, UNITED STATES

## Abstract

Chemical-genetics (C-G) experiments can be used to identify interactions between inhibitory compounds and bacterial genes, potentially revealing the targets of drugs, or other functionally interacting genes and pathways. C-G experiments involve constructing a library of hypomorphic strains with essential genes that can be knocked-down, treating it with an inhibitory compound, and using high-throughput sequencing to quantify changes in relative abundance of individual mutants. The hypothesis is that, if the target of a drug or other genes in the same pathway are present in the library, such genes will display an excessive fitness defect due to the synergy between the dual stresses of protein depletion and antibiotic exposure. While assays at a single drug concentration are susceptible to noise and can yield false-positive interactions, improved detection can be achieved by requiring that the synergy between gene and drug be concentration-dependent. We present a novel statistical method based on Linear Mixed Models, called CGA-LMM, for analyzing C-G data. The approach is designed to capture the dependence of the abundance of each gene in the hypomorph library on increasing concentrations of drug through slope coefficients. To determine which genes represent candidate interactions, CGA-LMM uses a conservative population-based approach in which genes with negative slopes are considered significant only if they are outliers with respect to the rest of the population (assuming that most genes in the library do not interact with a given inhibitor). We applied the method to analyze 3 independent hypomorph libraries of *M*. *tuberculosis* for interactions with antibiotics with anti-tubercular activity, and we identify known target genes or expected interactions for 7 out of 9 drugs where relevant interacting genes are known.

## Introduction

Chemical-genetic interactions (CGIs) represent cases where the sensitivity of a bacterial organism to an inhibitory compound is affected by changes in the expression level (either knock-down or over-expression) of a gene [1]. Such interactions could implicate the gene as a potential target of the inhibitor, or possibly that it is in the same pathway, or involved in a functionally interacting pathway. Hence, chemical-genetic experiments can be useful for antibiotic drug discovery, such as by profiling whole-cell growth inhibitors in high-throughput screens against a library of mutant strains (hypomorphs) with depletion of a subset of essential genes. Hypomorph libraries can be generated by a variety of technologies, ranging from promoter replacement (e.g. Tet-inducible promoters) [2], to ClpXP-mediated proteolytic degradation system (tagging genes with C-terminal DAS peptides) [3, 4], to CRISPRi (transcriptional blockade) [5]. Typically, a set of essential genes is selected, and each is knocked down individually in a separate clone, producing growth impairment (possibly to different extents). Then the culture is treated with an inhibitor (typically at a sub-MIC level). By utilizing unique nucleotide barcodes in the sequence constructs, the relative abundances of the mutants in the library can be profiled efficiently using next-generation sequencing by PCR-amplification of the barcodes. While the library as a whole experiences growth impairment due to the presence of the antibiotic, mutants with reduced levels of protein products of genes that interact with the compound, such as the target of the drug, could exhibit excess depletion, and such gene-drug pairs are identified as CGIs. This effect results from the synergy between the fitness effects due to the drug pressure and depletion of an essential gene which happens to be involved in the mechanism of action of the drug. For example, if the expression level of the target of a drug is depleted, then that clone might become hyper-susceptible to exposure to that drug, relative to the rest of the population. Depletion of other genes, such as those in the same pathway, or genes involved in drug resistance can also exhibit synergies with antibiotic treatment.

Analysis of chemical-genetics (C-G) data is similar to–but distinct from–methods for quantifying genetic (gene-gene) interactions (e.g. epistasis, super-additivity of fitness effects [6, 7]) and pharmacological (drug-drug) interactions (e.g. Chou-Tulaly Combination Index, [8]). The statistical analysis of a C-G experiment is aimed at identifying the gene(s) in the library that interact with a drug by looking for those with excess depletion (or enrichment) of barcode counts compared to the rest of the population. Quantifying statistical significance is important, because the genes can always be ranked by their apparent level of depletion, and there will always be a "most-depleted" gene, but this does not necessarily mean it is genuine functional interaction; they must be distinguished from random variations caused by spurious fluctuations or errors in estimation of the gene abundances in the library. The challenge of analyzing C-G data is that there are many sources of noise in the data (which takes the form of barcode counts from sequencing). The original abundances in the library are only approximately known, and the growth under different treatments and DNA extraction/preparation for sequencing are stochastic, resulting in variability due to both biological reasons and sampling error. Although each mutant might experience a certain level of growth impairment due to depletion of an essential gene, as well as drug treatment, there is inevitably going to be some experimental variance in apparent gene abundances between drug concentrations. Finally, not all genes might be represented in the library at equivalent levels a priori, and genes that are low-abundance to begin with (or as growth-impairment increases) become more difficult to reliably estimate as counts approach zero.

In previous work, Johnson et al [9] described an approach to statistical analysis of C-G data based on a generalized linear model (GLM), called ConCensusGLM (part of the PROSPECT methodology). Specifically, they fit gene abundances (normalized barcode counts) to a linear model (using the Negative Binomial distribution with a log-link function as a likelihood), with drugs (at different concentrations) as covariates. The GLM approach captures the dependence of gene abundances on drug treatments through coefficients in the linear model. To determine which interactions are statistically significant, the authors applied a Wald test, employing strain-wise dispersion estimates (from growth in DMSO controls). A Wald test evaluates whether a coefficient in the model is significantly different from zero [10]. However, the linear model in the ConCensusGLM pipeline treats each concentration independently (equivalent to single-point assays). The only dependence on drug concentration is between individual concentrations and the no-drug control (effectively estimating log-fold-changes), and thus it does not take advantage of the expected relationship between different doses, potentially making it more susceptible to random fluctuations. Furthermore, treating each concentration separately unnecessarily inflates the number of tests and therefore reduces the power of the analysis.

In this paper, we propose a new approach, called CGA-LMM, in which drug concentration is treated as a quantitative variable (covariate) in the linear model. The effects of drug concentration on a gene’s abundance is captured by a single coefficient (a slope) that incorporates information across multiple concentrations. Genes that interact with an inhibitor (directly or indirectly) are expected to show a synergistic relationship between drug concentration and protein depletion. At low drug concentrations, their abundance is expected to be similar to the rest of the population. However, as the drug concentration increases, the abundance of these mutants should begin to decrease, particularly at concentrations approaching the MIC. Our approach to assessing interactions is to calculate a slope of each gene’s abundance with respect to the drug concentration, integrating information across a range of concentrations and capturing systematic changes in the counts. This approach is expected to be more robust because it depends on trends observed over multiple conditions, and hence is less sensitive to random fluctuations of gene abundance at any individual concentration. Because depletion of different genes might cause different degrees of cellular growth impairment [4, 11], it is hard to predict the concentration at which the depletion will occur, and not all genes follow a perfect inhibition curve that is typical of an ideal dose-response relationship. However, if the abundance drops off at some point within the range of concentrations evaluated, the overall decrease in abundance would still exhibit a negative slope, thus allowing this approach to identify such cases.

Our CGA-LMM approach is implemented as a linear mixed model (LMM), where the fixed effects represent the average trend in the population, and the slope of each gene is represented as a conditional random effect (conditioned on drug concentration), so each gene can have its own unique slope. The aim of the approach is to identify genes that exhibit negative slopes that are significantly different than the rest of the population. We do this by using outlier analysis to test for genes with slopes that are outliers relative to the distribution of slopes over all genes in the library. This differs from testing whether a slope is statistically different from zero. The rationale behind using an outlier analysis is that there are multiple sources of noise in C-G experiments that are difficult to model explicitly, resulting in excess dispersion in the distribution of slopes. Failing to account for these sources of dispersion is likely to produce many false positives. Instead, we take an empirical view that each individual gene might have a slightly positive or negative slope. We determine this variance in the random effects post-hoc and use it to identify genuine CGIs, which must stand out from the rest of the population as *outliers* in the context of all the other genes. This is a more conservative approach that produces a shorter list of candidate interactions, but as we show, enriches for known interacting genes.

## Methods

### Linear mixed model for chemical-genetic interactions

We use a linear mixed model (LMM) to capture the relationship between gene abundances and drug concentrations (using log-transformations of both). Mixed-effect models (with fixed and random effects) are useful when there are subgroups of data that might differ from a general trend in an idiosyncratic way. In analysis of chemical-genetics data, we desire to use linear regression to capture the (fixed) effect of how abundance of knock-down mutants in the library is affected by increasing drug concentrations. However, depletion mutants for each gene might respond in a different way to increasing concentrations of the drug, which is key to distinguishing interactors vs. non-interactors. The random effects in the LMM capture the gene-specific abundances (intercepts) and concentration-dependence (slopes), which are themselves (as coefficients) assumed to be drawn from a Normal distribution of unknown variance. The linear mixed model is expressed as:

Y=XB+ZU+e

where, ***Y*** is the vector of the observed gene relative abundances (normalized barcode counts), and the vector of errors $e∼N(0,\sigma_{err}^{2})$ are assumed to be normally-distributed with some variance $\sigma_{err}^{2}$. Given *n* observations, ***X*** is a *n*×2 design matrix, with a column encoding the log_2_ of the concentration and a constant (1) representing the intercept. For control conditions (i.e., where no drug is used), a value two times lower than the minimum concentration is used, so that it appears as the lowest concentration value in the regression. The coefficients ***B*** are the fixed effects which will be fit in the model, representing an average slope and intercept for a given drug treatment (independent of gene). The ***ZU*** term represents random effects for capturing the gene-specific effects. ***Z*** is a *n*×2*g* matrix of covariates with *g* binary columns that encodes the information about which gene and concentration each observation represents. ***U*** is a 2*g*×1 matrix of random effects, including a slope and intercept for each gene. The random effects for each gene are assumed to be drawn from a higher-level multivariate Normal distribution, ***U***~*MVN*(**0**, **Σ**).

The unconditional variance for the fixed-effects part of the model, ***Y*** = ***XB*** (independent of random effects), can be decomposed into the overall variance of the model (residuals, ***e*** = ***Y***−***XB***−***ZU***, conditioned on both fixed and random effects), plus the variance of the predicted offsets due to the random effects (assuming they are uncorrelated):

d=Y−XB=e+ZU

$$var(d)=\sigma_{d}^{2}=var(e)+var(ZU)=\sigma_{err}^{2}+\sigma_{re}^{2}$$

The term for the variance of the random effects for each observation can be related to the variance ***Σ*** in the multivariate Normal distribution for the random effects vector ***U*** as

$$\sigma_{d}^{2}=\sigma_{err}^{2}+Z\Sigma Z’$$

where the ***ZΣZ***’ term projects the variance of the relevant random effects onto the individual observations based on their covariates. This can be rewritten as

$$\sigma_{d}^{2}=\sigma_{err}^{2}(I_{n}+ZDZ’)$$

where $D=\frac{1}{\sigma_{err}^{2}}\Sigma$ is a scaled covariance matrix among the random effects.

Using these definitions, the model can thus be fit with respect to the fixed and random effects by considering that *E*[***Y***] = ***XB*** and the $var(Y-XB)=\sigma_{err}^{2}H$ where ***H*** = ***I***_***n***_+***ZDZ***′. If the covariances of the gene-specific parameters Σ, and thus ***D*** and ***H***, were known, the maximum likelihood estimates of ***B*** and ***U*** could be estimated as [12]:

$$\hat{B}={\left(X\prime H^{-1}X\right)}^{-1}(X\prime H^{-1}Y)$$

$$\hat{U}=DZ\prime H^{-1}(Y-X\hat{B)}$$

Instead, we use restricted maximum likelihood (REML) to solve the system by an iterative procedure [13], as implemented in the *lmer* function in the *lme4* package in R. The resulting model then has estimates of the slope coefficients for each gene (random effects), along with variances. In the formula used to specify the model to *lmer*, Y ~ 1+conc+(1|gene)+(0+conc|gene), the fitting of the gene-dependent random effect parameters (intercepts and slopes) is intentionally decoupled, because we expect that the gene-specific interaction of the gene with the drug (slope) should be uncorrelated with the overall abundance of the gene in the library (intercept) [14], and this decoupling has been shown to produce more reliable estimates of random effect parameters when they are uncorrelated [15].

We are primarily interested in genes with negative slopes (i.e. genes whose depletion mutants decrease in relative abundance with increasing concentrations of the drug), as this synergy represents sensitization and hence potential interactions with the drug. However, genes with positive slopes could be informative too (i.e. genes where depletion confers a growth advantage, and hence selection, in the presence of the drug). To determine which genes most likely to represent C-G interactions (positive or negative) for a given drug, we compare it to the rest of the population to look for outliers. This differs from a Wald test [10], which would identify genes whose slope is statistically different from zero. Due to the multiple unaccounted-for sources of noise in C-G experiments, there are various reasons why the slopes of some genes might be different from zero, resulting in dispersion in the distribution of slopes. A robust model should account for such variability in order to avoid generating false positives. To address this, we take an empirical view that the average gene (which is assumed not to interact with the drug) might have a slightly positive or negative slope, and we determine this variance post-hoc. True interactors (CGIs) must stand out from the spread of the population as outliers. Thus, our test for statistical significance takes advantage of the distribution of random effects to evaluate which genes have slopes that are outliers. In contrast to a test which evaluates the significance of each coefficient in isolation, our method identifies genes as significant only in the context of all the other genes.

Outlier slopes were determined using a robust version of Z-scores for the random effect coefficients, called Zrobust, and look for outliers with respect to the population. Conceptually, we want to model the population of slopes as a sample from a Normal distribution, with a central tendency (mean) and dispersion (variance), and use Z-scores to determine which genes have the most extreme values. However, outliers could throw off estimates of these sufficient statistics. Zrobust (also called Modified-Z; [16]) is similar to a Z-score for samples from a Normal distribution, except it substitutes the median for the mean, and mean absolute deviation (MAD) for the standard deviation, which are better estimates of the sufficient statistics that are less sensitive to the influence of outliers. The Zrobust score for gene *i* is defined as:

$${Zrobust}_{i}=\frac{{0.6745*(s}_{i}-med(s))}{median(|s_{i}-med(s)|)}$$

where *s*_*i*_ is the slope of the gene (concentration-dependent random effect) estimated by the linear mixed model, and *med*(*s*) is the median of the population of slopes over all genes in the hypomorph library. The factor of 0.6745 is included to adjust med(s)±Zrobust to correspond approximately to the 25% and 75% quartiles (interquartile range). Iglewicz and Hoaglin suggest using |Zrobust|>3.5 as a cutoff to define outliers, which we adopt to identify genes in a hypomorph library that have the strongest evidence for C-G interactions with a given drug.

#### Hypomorph library preparation and sequencing

To evaluate our statistical method, we generated a hypomorph library of ClpXP-mediated depletion mutants for 162 essential genes in *M*. *tuberculosis* H37Rv by allelic replacement, where the native copy of each gene was deleted, and a C-terminal DAS-tagged copy was integrated into the L5 phage attachment site in the *M*. *tuberculosis* genome, as described in [17]. The *sspB* gene, needed for recognition of DAS-tagged proteins and targeting for proteolytic degradation through the native caseinolytic ClpXP protease, was also integrated at the L5 site and controlled by a “reverse” Tetracycline repressor that represses expression in the presence of anhydrotetracycline (ATC) [18]. Removal of ATC, allowed expression of *sspB*, which lead to the degradation of target protein through ClpXP. To achieve non-lethal doses of protein degradation, the levels of *sspB* expression were regulated through promotor variations [9, 17, 19, 20].

Single strain cultures were grown to mid-log phase for 7 days in Middlebrook 7H9 medium supplemented with 10% ADN, 0.5% glycerol, 0.05% Tween80, 25μg/ml streptomycin and 500ng/ml ATC at 37C with 5% CO_2_. ATC was replenished on 4th day of preculture.

For drug-exposure experiments, equal amounts of strains were combined based on optical density. The combined culture was washed twice with Middlebrook 7H9 medium supplemented with 10% ADN, 0.5% glycerol, 0.05% Tween80 to remove ATC and diluted to a starting OD of 0.01 for inoculation of 48-well flower plates (M2P labs, catalog number MTP-48-OFF) with 1ml culture volume per well. Wells were sealed with gas-permeable foil. The mixed culture was exposed to drug for 14 days, incubated at 37°C and 5% CO_2_. Concentrations tested for each drug ranged from 0.125x to 1.0x MIC, following 2-fold dilutions, plus a no drug control, with 6 replicates each. MICs for the six drugs were: levofloxacin = 0.18 μg/ml, moxifloxacin = 0.14 μg/ml, isoniazid = 0.022 μg/ml, fidaxomycin = 0.29 μg/ml, bedaquiline = 0.58 μl/ml, sulfamethoxazole = 6.0 μg/ml. Compounds were dispensed randomly, and wells were normalized, so that each would contain 1% DMSO.

For DNA extraction 100μl of each well was heat inactivated at 80C for 2h. 300x resuspended lysate was combined with equal volume of 25% DMSO. For barcode amplification Q5 hot start polymerase (NEB) was used. Per 20μl PCR reaction 4ul Q5-buffer, 0.5μl dNTPs (10mM each), 0.1μl Q5 hot start polymerase, 2μl of per primer are combined with 8ul lysate. The primers contain sequences for Illumina sequencing as well as additional barcodes to encode plate and well. PCR reaction started with 2 min 98°C, then 22 cycles of 98°C for 10 sec, 50°C for 20 sec, 72°C for 20 sec, followed by final extension for 2 min at 72°C.

10 μl PCR reaction were combined to a total volume of 2.2ml. To remove PCR reagents, AMPureXP magnetic beads from Beckman Coulter were used following their protocol. To further clean the amplicons, Pippin gel extraction technology (Sage science) was used to excise DNA fragments with a length of 200bp. The PCR products were sequenced using Hiseq4000 with single read 50 with 20% PhiX, resulting in a median of 505,000 reads per individual sample (replicate), after demultiplexing. There were 6 replicates for each drug concentration. Read-counts were determined by extracting barcodes from the sequencing reads and tabulating counts for each gene in each sample, according to the nucleotide barcode assignments designed into the adapter sequences above. Plate id barcodes (8 bp) were extracted from nucleotides 1 through 8 of read 1 (allowing up to 1 mismatch), strain barcodes (10 bp) in nucleotides 25 through 35 of read 1. Well id barcodes (7 bp) were encoded in read 2, which was used to demultiplex the samples in each lane.

#### Copper growth inhibition screen

A separate pooled hypomorph library with mutants of 339 essential genes was generated as described above. This hypomorph library was constructed with *sspB* expressed from a weakened Ptet promoter (P766) containing an inserted tetO_4C5G_ sequence between the -10 and -35 regions. As described above, the *sspB* gene integrated at the L5 phage site in the H37Rv genome also carries the reverse TetR38 repressor, allowing for limited expression of *sspB* (and thus moderate levels of target degradation) in the absence of anhydrotetracycline (see [3], for a review). The sequence of the modified P766 promoter is available upon request. The library was washed twice in PBS supplemented with 0.1% Tyloxapol and resuspended in minimal medium supplemented with 0.1% Tyloxapol and 0.1% glycerol, 0.1% acetate, or 0.1% cholesterol at OD600 of 0.05 for inoculation to 96-well plates. Minimal medium was made as previously described but with ferric chloride (100 μM) replacing ferric ammonium citrate [21]. 5 replicates were prepared for each condition. Copper sulfate solution was added to the top row of wells at 1–8 μM in 2-fold serial dilutions below the MIC of 16 μM we measured. A no-copper growth condition was also included for each carbon source. Libraries were grown statically for 2 weeks at 37 degrees. Upon completion, 96-well plates were heat-inactivated at 85 degrees for 2 hours. Barcodes were PCR amplified as described previously [9]. Individual libraries were mixed with 1:1 20% DMSO and heated for 10 minutes at 98 degrees prior to multiplex PCR reaction. Amplified barcodes were purified using SPRI-based purification methods and sequenced using an Illumina NextSeq 550 with single-ended reads for 75 cycles and 1% PhiX as a control. Each replicate consisted of approximately 1 million reads. Sequencing reads were processed using Bowtie software package, with index mismatch set to 2 bases and barcode mismatch set to 1 base, and barcodes for each gene were tabulated.

#### Data pre-processing

After the sequencing data is obtained, the reads are de-multiplexed and formatted into a matrix (spreadsheet) *C*_*g*,*s*_ containing counts for each gene of the hypomorph library in each sample. The metadata for each sample includes drug, concentration, and possibly other data that could be used as covariates (number of days of incubation, carbon source in medium, *sspB* promoter strength, etc.). Genes or samples with less than an average of 100 barcode counts per observation were filtered out, as well as genes with >10% relative abundance at any concentration. Finally, the counts are normalized to produce relative abundances by dividing each the observed count for each individual barcode by the total counts for that sample. This step is done to adjust for the different numbers of reads sequenced for each sample, represented as another matrix *A*_*g*,*s*_, where the values range between 0 and 1 (e.g. fractions of the population). The matrix of abundances is melted into a column matrix of all relative abundances, *Y*_*n*×1_, (where *n* = *g*×*s*), along with drug treatment and log_2_-concentration for each observation in a parallel matrix of covariates, ***Z***, which is used in the statistical modeling.

## Results

### Analysis of chemical-genomic interactions in a previous screen of an *M*. *tuberculosis* hypomorph library

We evaluated our CGA-LMM model on chemical-genetics analysis data previously published by [9] (downloaded from www.chemicalgenomicsoftb.com). (A summary of all datasets analyzed in this paper is provided in S1 Table, with library source, number of genes, concentration ranges for each drug, MICs, total barcode counts, etc.) This dataset includes the raw read counts of a hypomorph library (library 1) consisting of 152 ClpXP-mediated knock-down mutants of essential genes in Mtb treated with the following inhibitors over a range of concentrations–rifampin, trimethoprim, methotrexate, and BRD-4592. These anti-tubercular drugs are known to inhibit the pathways of translation, folate synthesis (trimethoprim and methotrexate), and tryptophan synthesis, respectively. Johnson et. al [9] analyzed this dataset using a generalized linear model approach called ConCensusGLM, which compared the mutant abundances at each concentration independently to the no-drug control. They observed that relevant target genes were significantly depleted for each drug. However, ConCensusGLM labeled almost all of the genes (≥137 out of 152) in the hypomorph library as significant interactions (Table 1). We applied our LMM-based approach to this dataset and found similar results, yet our solution is more conservative in terms of identifying only the top 4 interacting genes as outliers for each drug (see slopes and Zrobust scores for all genes and drugs in S2 Table). For comparison, we also performed simple linear regressions of the relative abundance against log-concentration for each gene and tested the significance of the slope coefficient, in the sense of being different from 0, based on the *t*-statistic. As shown in Table 1 (and in S2 Table), many genes (up to 64 out of 152) have significantly negative slopes in this sense for each drug, but the outlier analysis focuses attention on only a small subset of genes with the most extreme slopes that stand out from the population.

**Table 1:** Summary of significantly interacting genes from the CGA-LMM model, simple linear regression, and the ConCensusGLM model (Johnson et al, 2019).

		CGA-LMM		Linear Regression	ConCensus GLM	
Drug	Expected target	Number of significant genes (Zrobust < -3.5)	Rank of expected target (out of 152)	Number of genes with negative slope significantly different than 0 † (out of 152)	Number of significant genes** (out of 152)	Rank of expected target (out of 152)
Trimethoprim	*trpG* *	4	#2	30	138	#15
Methotrexate	*trpG* *	4	#1	15	145	#3**††**
Rifampin	*rpoB*	4	#14	64	148	#3**†††**
BRD-4592	*trpA*	4	(not in hypomorph library)	37	152	------

*TMP and MTX bind to and inhibit DHFR. However, the Johnson et al (2019) study showed that *trpG* has a chemical-genetic interaction with antifolate drugs.

† test of t-statistic, adjusted P-value<0.05 using Benjamini-Hochberg procedure for multiple test correction

**significance defined as minimum p-value < 1e-10 over same concentrations as analyzed for CGA-LMM, and mean log-fold-change<0

†† tied with 6 genes with Pvalue = 0; *trpG* is ranked 3^rd^ based on mean log-fold-change

††† tied with 49 genes with Pvalue = 0; *rpoB* is ranked 3^rd^ based on mean log-fold-change

**Fig 1:**
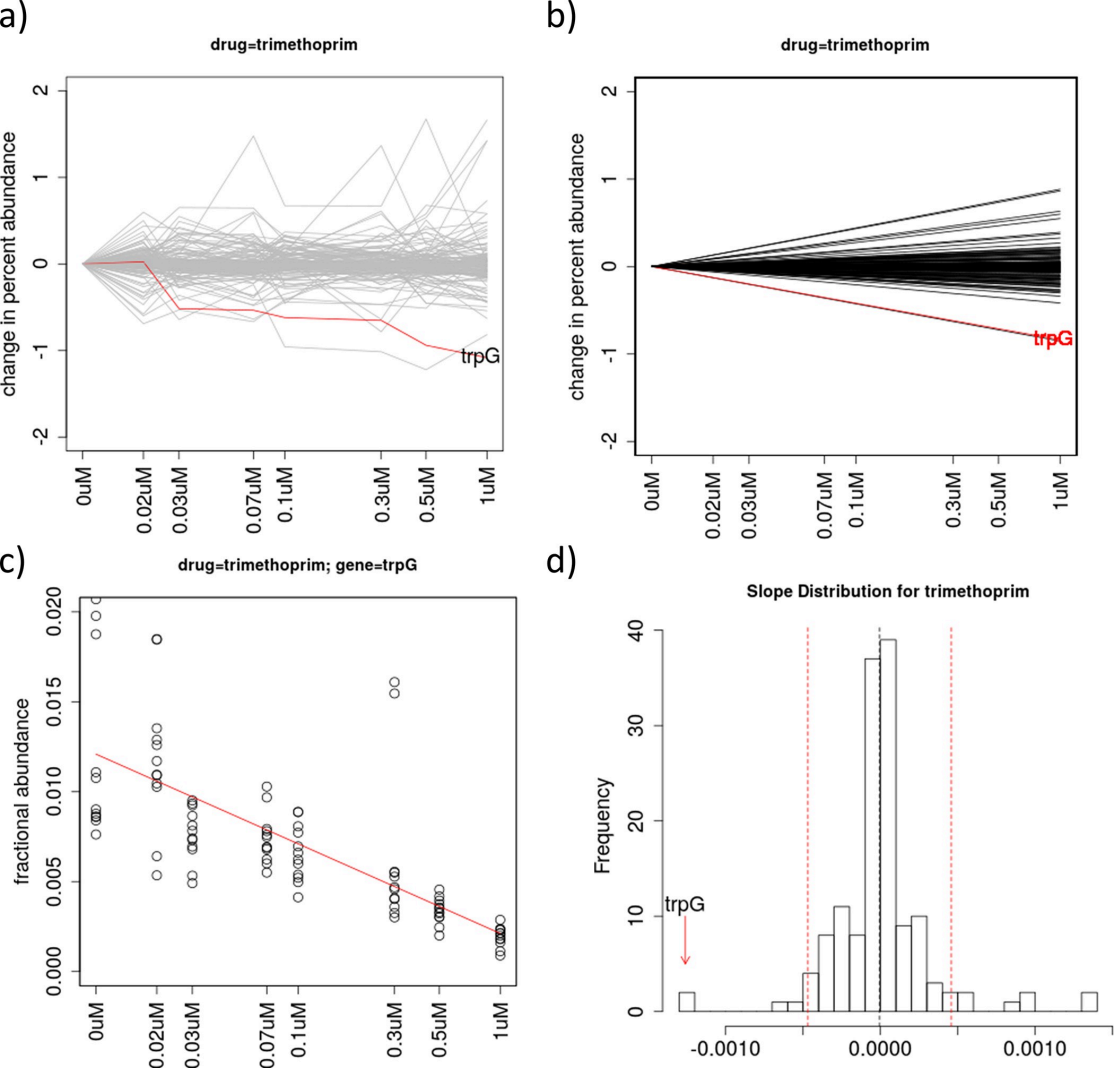
Analysis of data for treatment of an *M*. *tuberculosis* hypomorph library with trimethoprim. (a) Plot of relative abundances of knock-down mutants for all 162 genes in the library, with the known interacting gene *trpG* highlighted in red, showing depletion as concentration increases. The Y-axis represents the change in percent abundance of each gene, which is calculated by subtracting the mean relative abundance for each gene at 0 μM, so they all fan out from 0 in the superposition. (b) Plot of regression lines for all the genes based on slopes as random-effect coefficients in the linear mixed-model, fit to the abundance data in (a). (c) Plot of relative abundance data points and regression line fit specifically for *trpG*, as an illustration of the variability of the data among concentrations and replicates. (d) Histogram of the slope coefficients for all genes in the library, with *trpG* highlighted as an outlier (most negative slope). The dashed red lines indicate the outlier cutoffs defined by Zrobust = ±3.5.

Trimethoprim (TMP) inhibits *dfrA* (dihydrofolate reductase, DHFR) in the folate synthesis pathway. CGA-LMM analysis of this dataset identified 4 genes with outlier negative slopes that potentially interact with TMP. In contrast, ConCensusGLM reported many more interacting genes (137 out of 152). The top-ranked gene with most significant negative slope was *lipA* (Table 2), whose relevance to trimethoprim sensitivity is unknown. Although *dfrA* did not exhibit a negative slope in this experiment, the gene with the 2^nd^ most negative slope (Zrobust = -9.5) was *trpG* (Fig 1), consistent with its role at the branch-point of the folate pathway (see Discussion). This interaction was also observed by Johnson et. al [9]. (Gene abundance plots and slope histograms for all drugs analyzed in this paper are provided in S1 File.)

**Table 2:** Genes interacting with four drugs in the Johnson et al (2019) dataset (with outlier negative slope in CGA-LMM model and Zrobust<-3.5).

drug	Significantly interacting genes (slope<0, Zrobust<-3.5) (rank # out of 152 genes in hypomorph library)	Zrobust
trimethoprim	#1: *lipA*—lipoyl transferase	-9.6
	**#2: *trpG*—aminodeoxychroismate synthase**	-9.5
	#3: *desA1*—fatty acid desaturase	-4.4
	#4: *nusA*–transcription termination/antiterm. protein	-3.8
methotrexate	**#1: *trpG*—aminodeoxychroismate synthase**	-6.0
	#2: *gyrA*—DNA gyrase	-4.8
	#3: *ftsK*—ATPase involved in cell division	-4.2
	#4: *gca*—GDP-mannose dehydratase	-3.0
rifampin	#1: *dapF*—diaminopimelate epimerase	-6.4
	#2: Rv3267	-6.1
	#3: *desA1*—fatty acid desaturase	-4.3
	#4: *acn*—aconitase	-3.9
	. . .	---
	**#14: *rpoB*—RNA polymerase beta subunit**	-1.7 (n.s)
BRD-4592	#1: *desA1*—fatty acid desaturase	-5.0
	#2: *ftsK*—ATPase involved in cell division	-4.3
	#3: Rv3267	-4.1
	#4: *dapF*—diaminopimelate epimerase	-3.9

Out of a library of 155 genes, there were 4 genes with outlier negative slopes for each drug. Genes relevant to the mechanism of resistance are bold-faced. Note that *rpoB* was ranked highly for rifampin, though it did not exceed the Zrobust cutoff (n.s. = not significant). Also, the expected target of BRD-4592, *trpA*, was not represented in the hypomorph library.

Methotrexate (MTX) also targets *dfrA* in the folate pathway. CGA-LMM analysis again yielded a much smaller number of significant interactions for MTX compared to ConCensusGLM (4 genes out of 152 in the hypomorph library, versus 145 significant genes identified by ConCensusGLM). *trpG* was ranked as the top gene with the most negative slope (Zrobust = -6.0).

**Fig 2:**
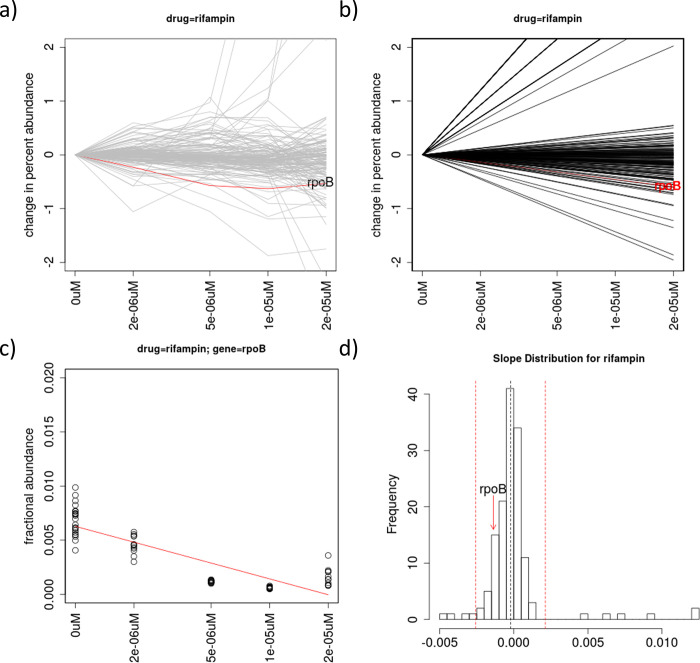
Analysis of data for treatment of an *M*. *tuberculosis* hypomorph library with rifampin. (a) Plot of relative abundances of knock-down mutants for all 162 genes in the library, with the known interacting gene *rpoB* highlighted in red, showing depletion. The Y-axis represents the change in percent abundance of each gene, as in Fig 1. (b) Plot of regression lines for all the genes based on slopes as random-effect coefficients in the linear mixed-model. (c) Plot of relative abundance data points and regression line fit specifically for *rpoB*. (d) Histogram of the slope coefficients for all genes in the library, with *rpoB* highlighted (with negative slope, but not an outlier). The dashed red lines indicate the outlier cutoffs defined by Zrobust = ±3.5.

Rifampin (RMP) binds to and inhibits *rpoB*, the beta-subunit of the RNA polymerase. CGA-LMM analysis identified 4 genes that met the significance cutoff for outliers with negative slopes (Zrobust<-3.5). In contrast, ConCensusGLM identified 148 genes out of 152 as significantly interacting with RMP (Table 1). *rpoB* is tied with 49 genes with a P-value of 0.0, which masks the significance among so many alternative possible interactions. (However, by breaking ties based on mean log-fold-change, *rpoB* has the 3^rd^ most negative LFC.) The gene that shows the most significant depletion in our concentration-dependent analysis was *dapF* (Zrobust = -6.4), which is involved in diaminopimelate synthesis (Table 2). While *rpoB* was not categorized as an outlier (Zrobust = -1.7) in the CGA-LMM analysis of this dataset, it has a negative slope that is statistically significant (in the sense of being different from 0 based on the t-statistic of the regression coefficient in a linear model, adjusted p-value = 1.3e-20) and is ranked the 14^th^ most-depleted gene out of 152 genes in the library (Fig 2). Thus, although the *rpoB* mutant shows depletion as RMP concentration increases, the effect was not as extreme as for several other genes. Johnson et al. [9] also has a similar observation, in terms of *rpoB* being depleted in presence of rifampin, but the depletion was muted compared to several other genes.

The target of BRD-4592 is *trpA*, required for tryptophan biosynthesis [22]. However, this gene was not present in the dataset available for the hypomorph library. The CGA-LMM model yielded 4 significant interactions for this drug (with Zrobust<-3.5): *desA1*, *ftsK*, Rv3267, and *dapF* (Table 2), though there is no known connection of any of these to exposure to BRD-4592. In contrast, ConCensusGLM determined that all 152 genes had significant interactions with BRD-4592 (Table 1). Among genes in the tryptophan synthesis pathway, only *trpG* (which also has anthranilate synthase activity) is present in the hypomorph library, but it did not have an outlying slope (Zrobust = -0.39).

### CGA-LMM identifies expected interactions for anti-tubercular drugs with known mechanisms

To evaluate our statistical method on additional antibiotics, we constructed a new hypomorph library consisting of 162 essential genes in *M*. *tuberculosis* H37Rv and profiled it under exposure to 6 anti-tubercular drugs with well-understood mechanisms of action–levofloxacin, moxifloxacin, isoniazid, fidaxomicin, sulfamethoxazole, and bedaquiline–over a range of concentrations. The concentrations tested spanned a range of 0.125–1.0 times the MIC (minimum inhibitory concentration) in 2-fold dilutions (MICs for the six drugs are listed in Methods). Table 3 lists the outlier interactions (with Zrobust<-3.5) identified by CGA-LMM for the 6 drugs, and Table 4 summarizes the known targets and significant interactions for the six drugs used in our experiment (with the hypomorph library containing knock-down mutants for 162 genes). Raw barcode counts are provided in S3 Table; CGA-LMM results (with estimated slopes and robust Z scores) for all gene-drug interactions are provided in S4 Table.

**Table 3:** Interacting genes (with negative outlier slopes, Zrobust<-3.5) for six anti-tubercular drugs, out of a hypomorph library with 162 genes, ranked in order of most negative slope at the top.

	Levofloxacin	Moxifloxacin	Isoniazid	Fidaxomycin	Sulfameth-oxazole	Bedaquiline
	*asnB* *thyA* *dapB* gyrA* *iscS* *embC* *kasB* *dnaN* *Rv1836c*	*asnB* *thyA* *Rv1836c* *dapB* *kasB* *iscS* *embC* gyrA* *dnaN* *moxR1*	ino1* *thyA* *asnB* *iscS* *dapB* *menH* kasB* *nusA* *fadD30* *mmpL2*	*thyA* *asnB* *dapB* *kasB* *Rv1836c* rpoB* *metA*	*fas* *desA1* *iscS* *ino1* *dapB* *menH* thyA* *kasB* *ilvC* *mmpl2* *Rv1836c* *fadD30* *rpoB* *embC* *aspB* *Rv0260c* *aspS* *Rv0289* *gyrA* *nusA*	*Rv1836c* *Rv3267* *dnaN* *pstP*
# of candidate interactions (outliers) by CGA-LMM	9	10	9	7	20	4
# of genes with slope significantly <0 by t-test in linear model	61	22	9	43	33	76

Genes with slopes not significantly different from zero (Padj≥0.05) have been removed. Genes related to the mechanism of action are marked with an asterisk.

**Table 4:** Summary of interactions of relevant genes for various drugs in hypomorph library with 162 essential genes.

drug	# genes with significant negative interactions (Zrobust<-3.5, Padj<0.05)	known target of drug	genes related to drug mechanism of action #rank (Zrobust)
**levofloxacin**	9	***gyrA***	#4: ***gyrA*** (-4.6)—DNA gyrase
**moxifloxacin**	10	***gyrA***	#8: ***gyrA*** (-5.5)—DNA gyrase
**isoniazid**	9	*(inhA)* *	#2: ***ino1*** (-19.6)—inositol-3-phosphate synthase
			#11: ***kasB*** (-4.4)—ketoacyl synthase
**fidaxomicin**	7	***rpoB***	#9: ***rpoB*** (-5.0)—RNA polymerase beta subunit
**sulfamethoxazole**	20	*(folP1)* *	#7: ***thyA*** (-11.1)–thymidylate synthase
**bedaquiline**	4	*(atpE*)*	#9: ***atpB*** (-2.8†)—subunit of ATP synthase
			#13: ***atpH*** (-2.2†)—subunit of ATP synthase
			#23: ***atpF*** (-1.3†)—subunit of ATP synthase
			#38: ***atpG*** (-0.8†)—subunit of ATP synthase

† does not exceed the outlier cutoff of Zrobust<-3.5, but still has a negative slope showing synergy with the drug

* gene not represented in hypomorph library

**Fig 3:**
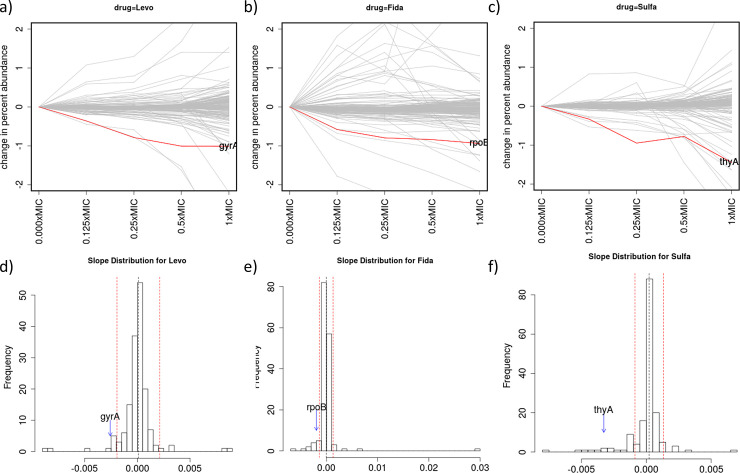
Analysis of chemical-genetic interactions with levofloxacin (Levo), fidaxomicin (Fida), and sulfamethoxazole (Sulfa). Abundance plots for all genes in the hypomorph library (a-c), and histograms of the distribution of slopes for each gene (d-f). *gyrA*, *rpoB*, and *thyA* clearly stand out as outliers for levofloxacin, fidaxomicin, and sulfamethoxazole, respectively.

For levofloxacin, 9 out of 162 genes are identified with outlier negative slopes using CGA-LMM (even though 61 genes had negative slopes that were significantly different from 0). *gyrA* (DNA gyrase subunit A, the expected target of fluoroquinolones) is the 4^th^ ranked gene, with a Zrobust of -4.6. The depletion effect with increasing inhibitor concentration is shown in Fig 3a, reflecting the chemical-genetic interaction between levofloxacin and *gyrA*. Moxifloxacin, another fluoroquinolone, has *gyrA* ranked 8^th^ among 10 genes with showing significant depletion (out of 162), with a Zrobust of -5.5. The top-ranked gene showing the most significant depletion for both levofloxacin and moxifloxacin was *asnB* (asparagine synthetase) (Table 3), whose relationship to fluoroquinolone resistance is unknown.

Isoniazid (INH) targets *inhA* (fatty acid enoyl-ACP reductase), which is in the FAS II pathway for synthesis of long-chain fatty acids and ultimately mycolic acids [23]. INH is a pro-drug that must first be activated to a radical by the KatG catalase, which then forms an adduct with NADH, which binds to and inhibits *inhA*. Thus, one might expect that depletion of *inhA* would be synergistic with INH treatment (causing barcode counts to decrease), and depletion of *katG* would be antagonistic (causing barcode counts to increase, representing enhanced survival due to depletion of an activator). However, neither *inhA* nor *katG* is in the hypomorph library. Using our CGA-LMM analysis, 9 genes are identified as interactions (Table 3), with negative slopes that are outliers (Zrobust<-3.5) with respect to the rest of the population of genes, after filtering out genes with slopes not significantly different from zero (Padj≥0.05). The most synergistic gene (rank #1) was *ino1* (inositol-1-phosphate synthase) (Zrobust = -31.4), which is involved in synthesis of mycothiol (see Discussion). Another interacting gene is *kasB* (β-ketoacyl synthase), which is in the fatty-acid/mycolic-acid synthesis pathway (FAS II cycle, like *inhA*), is ranked as #7 on the list of outliers.

Fidaxomicin binds to *rpoB*, the β subunit of the RNA polymerase, and inhibits transcription initiation [24]. Using our CGA-LMM analysis, 7 genes with significant slopes met the outlier cutoff (Zrobust<-3.5 and Padj<0.05). *rpoB* was ranked #6 on the list of significant interactions (Zrobust = -5.0). Fig 3B shows the concentration-dependence of *rpoB* mutants and the distribution of the slopes of all the other genes in the library when treated with fidaxomicin.

The target of sulfamethoxazole is dihydropteroate synthase (DHPS, *folP1*) in the folate pathway. *folP1* which was not present in the hypomorph library. CGA-LMM analysis identified 20 genes with interactions (negative slopes that are outliers; Table 3). The two top-ranked genes were *fas* (fatty-acid synthase) and *desA1* (fatty-acid desaturase), but the link between fatty-acid synthesis and sulfamethoxazole has not been established. *thyA* (thymidylate synthase) is ranked #7 on the list of outliers (Zrobust = -11.1), highlighted in Fig 3C. The only other genes of the folate pathway that are represented in the hypomorph library are *trpG* and *folB*, which are ranked highly (#26 and #32 out of 162), but did not exhibit enough depletion to meet the cutoff for outliers (Zrobust scores of -1.8 and -1.2). Although most of the focus of our CGA-LMM analysis has been on negative interactions; positive interactions can sometime also be informative. We note that, for SMX, the gene with the 2^nd^ most positive slope was *efpA* (Zrobust = +9.5), an essential efflux pump (see Discussion).

**Fig 4:**
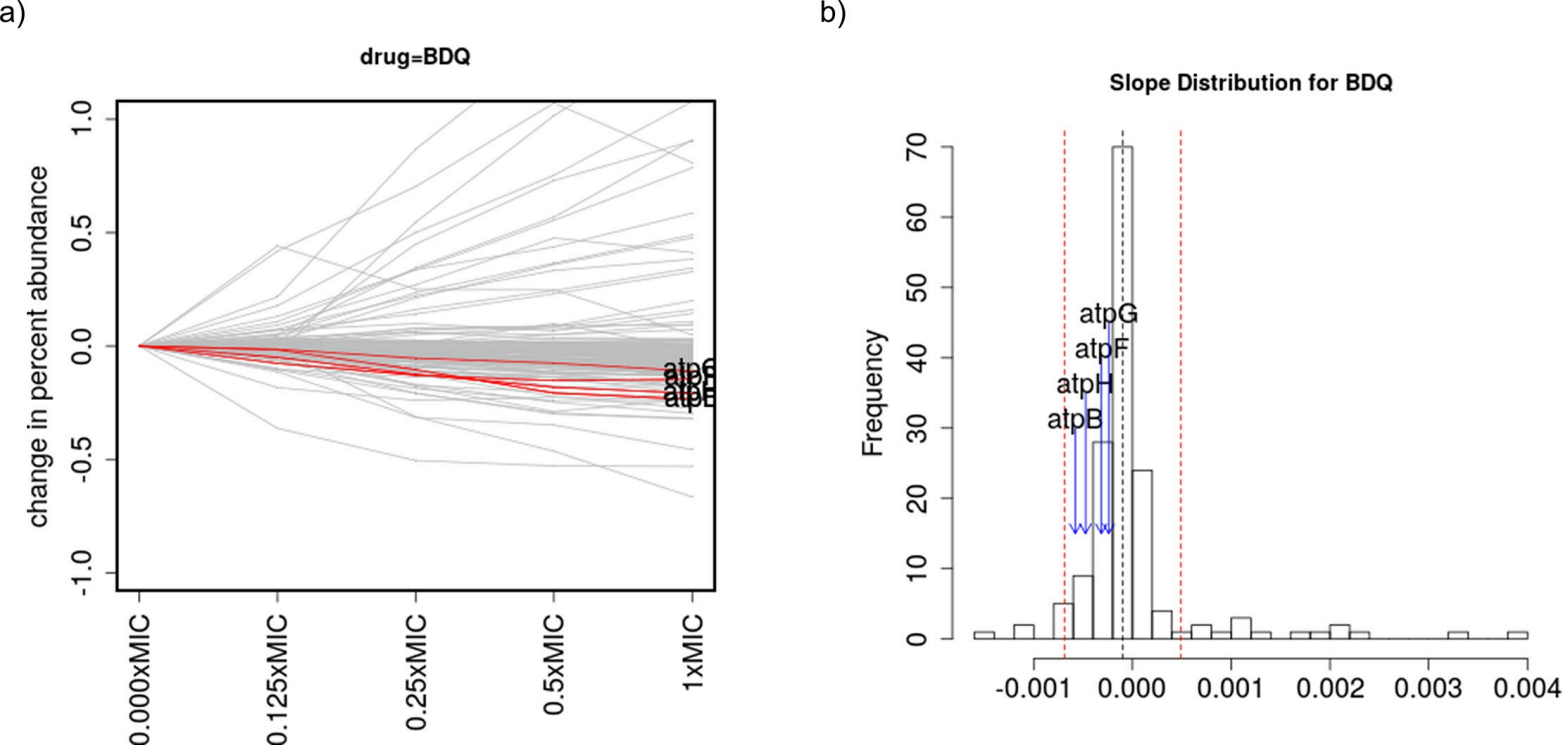
Analysis of chemical-genetic interactions with bedaquiline (BDQ). a) abundance plot for all genes in the hypomorph library, and b) histogram of the distribution of slopes for each gene. For bedaquiline, none of the 4 ATP synthase subunits in the library was detected as an outlier, but collectively, they all exhibited negative slopes, indicating depletion with increasing drug concentration.

Bedaquiline (BDQ) targets genes of the ATP synthase complex (4 out of 8 subunits are in the hypomorph library: *atpB*, *atpF*, *atpG*, and *atpH*). Specifically, BDQ binds to subunit C of the membrane complex [25] (which is annotated as *atpE* in the Mtb genome). Only 4 genes meet the significant cutoff for outliers with negative slopes. Although none of the 4 ATP synthase genes is an outlier on its own, they all have significant negative slopes (in the sense of being significantly different from 0, based on a *t*-test of coefficients in a linear regression, adjusted p-value<0.05, except for *atpF* which has Padj = 0.0504; S4 Table) and are ranked highly–#9, #13, #23, and #38 (out of 162 genes in the library) (Table 4)–indicating that knock-down mutants of these genes are all depleted in the library as BDQ concentration increases. This is evident from Fig 4, which shows a negative trend for these four *atp* genes as a group. Pathway analysis can be used to show that this systematic depletion with increasing drug concentration across all four ATP synthase genes is statistically unlikely. We used Gene Set Enrichment Analysis (GSEA, [26]) to analyze the 79 functional categories of genes in the annotation of the H37Rv genome (see Table 1 in [27]). Using GSEA (with algorithm parameter *p* set to 0), ATP Proton-Motive Force (I.B.8) is the only significantly enriched category of genes, with an adjusted p-value of 0.046.

### Chemical-genetic interactions with copper

**Fig 5:**
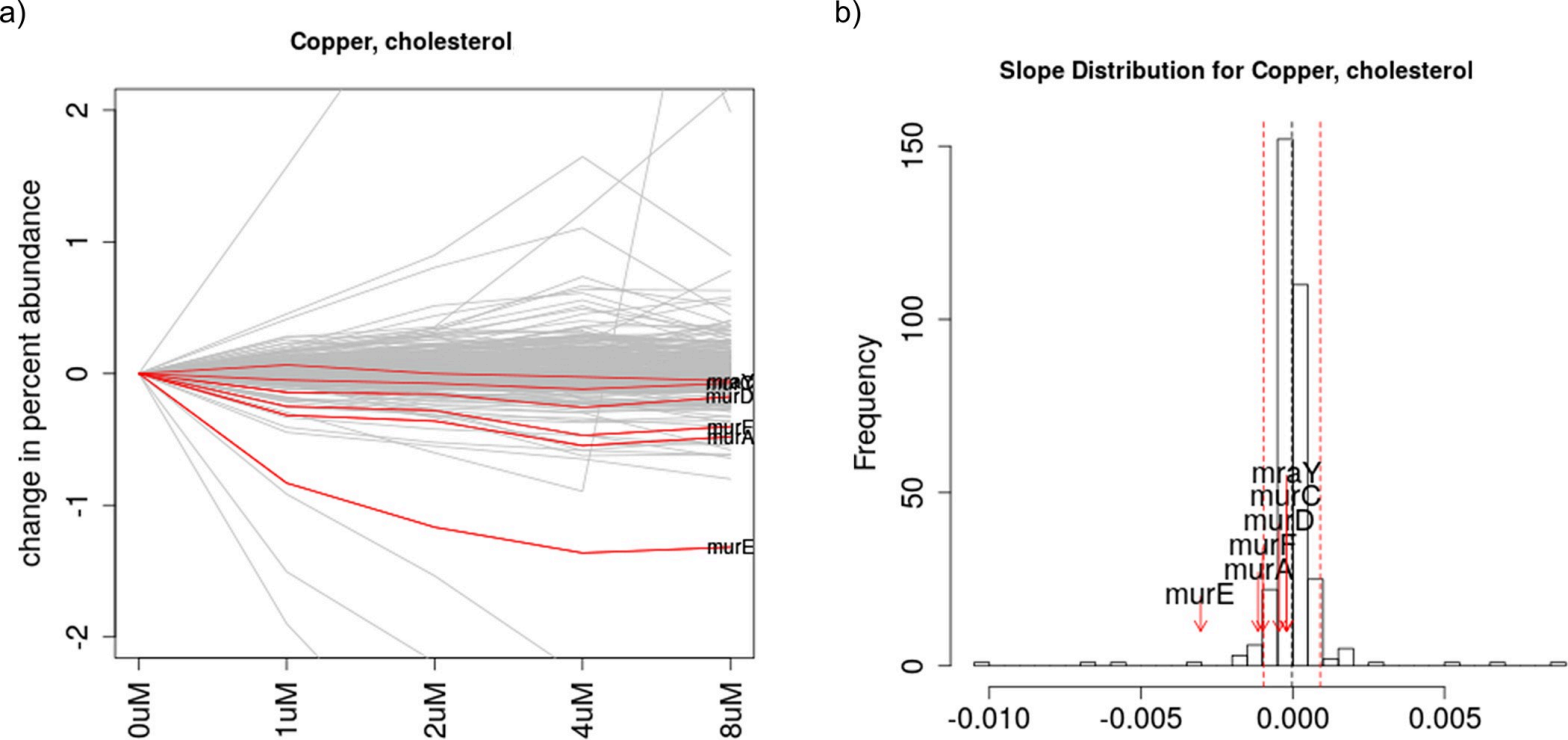
Abundance plot and slope histogram for genes in an *M*. *tuberculosis* hypomorph library treated with copper. Six genes of the muramic acid pathway, which is required for peptidoglycan synthesis, are highlighted in red, indicating increased sensitivity of this pathway with increasing concentrations of copper.

Copper is known to be bactericidal at high concentrations for many bacteria, and is believed to interfere with cell-wall maintenance [28, 29], as well causing other problems, including oxidative damage and mismetallation of various metalloenzymes [30]. To probe the genes in *M*. *tuberculosis* that interact with copper, we constructed a new hypomorph library with 339 essential genes (barcode counts are provided in S5 Table). We selected the library in the presence of varying concentrations of copper sulfate (1 to 8 μM, just below the MIC of 16 μM) on media with 3 different carbon sources–glycerol, acetate, and cholesterol. Analysis of the chemical-genetics data showed an average of 6–18 genes that interacted negatively with copper in each of the 3 conditions (carbon sources) (S6 Table). TrxB2 (thioredoxin reductase) was the top-ranked gene with most negative slope on all 3 carbon sources. In addition, pathway analysis yielded another insight: genes in the peptidoglycan (PG) pathway are frequently enriched. Analysis with GSEA shows that Murein Sacculus and Peptidoglycan is the only significantly enriched pathway among all the 79 functional categories [27], with an adjusted p-value of 0.028. In particular, 6 of the 7 genes in the PG pathway that are represented in the hypomorph library are involved in muramic acid synthesis *murA*, *murC*, *murD*, *murE*, *murF*, and *murX* (also known as *mraY*). The most striking case of the enrichment of *mur* genes as negative outliers is when cholesterol is used as a carbon source. In this case, CGA-LMM identified 14 genes out of 339 that had outlier negative slopes (decreasing abundance with increasing Cu concentration), including 3 *mur* genes: *murE* (ranked #4), *murA* (#12), and *murF* (#14) (Table 5). In addition, even though they were not categorized as outliers (Zrobust scores not below the outlier cutoff of -3.5), *murD*, *murC*, and *murX* also had negative slopes (ranked #39, #64, #71 out of 339) that were statistically significant, in the sense of being significantly less than 0 (via a test of the t-statistic of coefficients in a linear regression, adjusted p-value<0.05, see S6 Table), indicating all 6 *mur* genes show depletion. While some *mur* genes were ranked highly for growth on the other two carbon source (acetate and glycerol), the effect was less pronounced and was not significant (by GSEA analysis). Fig 5 shows the plot of the change in relative abundances of the genes in the hypomorph library when treated with copper in cholesterol, with *mur* genes highlighted.

**Table 5:** Analysis of C-G interactions for copper exposure.

	cholesterol	acetate	glycerol
# of genes evaluated in hypomorph library	339	339	339
# of genes with outlier negative slopes (Zrobust<-3.5)	14	6	18
# of Mur pathway genes with outlier negative slopes	3	0	1
rank of Mur genes	**#4 *murE***	#9 *murA*	***#8 murE***
	**#12 *murA***	#10 *murF*	#28 *murA*
	**#14 *murF***	#17 *murE*	#47 *murD*
	#39 *murD*	#21 *murD*	#53 *murF*
	#64 *murC*	#65 *murC*	#135 *murX*
	#71 *murX*	#91 *murX*	#175 *murC*
mean rank of 6 Mur genes (murACDEFX)	34.0	35.5	74.3
Adjusted P-value of Murein Sacculus and Peptidoglycan pathway by GSEA	0.020*	0.065	0.244

A hypomorph library of 339 essential genes in *M*. *tuberculosis* H37Rv was grown on 3 different carbon sources and exposed to increasing concentrations of copper. The 6 Mur genes are ranked most highly for cholesterol, including 3 genes ranked among the 14 with outlier negative slopes, and the enrichment for Mur genes when grown on cholesterol is statistically significant based on GSEA. Genes that are bold-faced have negative slopes that are outliers (Zrobust<-3.5).

## Discussion

In this paper, we have shown how linear mixed models can be used in the analysis of chemical-genetic interaction data to account for concentration-dependence. The concentration-dependence is represented by gene-specific slope coefficients (as random effects) that capture the change in relative abundance of each mutant in the library as drug concentration increases. Although the relative abundance for most knock-down mutants in the hypomorph library would be expected to be unaffected by increasing concentration of drug, the premise of the C-G experiment is that artificial depletion of essential genes that interact with a drug, would be expected to exhibit a negative synergy with the drug, analogous to drug-drug interactions [8], where those mutants are sensitized, leading to excess depletion relative to the rest of the population in a dose-dependent fashion. As demonstrated for several drugs with known targets (discussed below), the analysis enriches for known interactions, by requiring a decreasing (or increasing) trend over multiple concentrations, which deprioritizes mutants that reach significant depletion only at a single concentration (e.g. log-fold-change compared to no-drug treatment) which is not reinforced at other concentrations. In the approach implemented in CGA-LMM, candidate interactions are determined by genes with outlier negative slopes, which more stringent than asking which slopes are significantly different from zero. This approach was chosen to acknowledge that there are multiple sources of noise in these experiments that could cause arbitrary genes to have slopes differing from zero (slight increases or decreases). While the magnitude of these variances is unknown a priori, it will be evident in the empirical dispersion of the population of slopes post hoc. We used a robust Zscore (Zrobust) to identify candidate interactors as outliers that exhibit concentration-dependent depletion outside the typical range represented by the rest of the genes. As shown in Tables 1 and 3, for most drugs, there were many genes that had negative slopes significantly different from zero (as many as 76 out of 162 in one library), but only a small subset of these gene qualified as outliers, which provides a more focused list of candidate interactions.

Importantly, this method can potentially identify not only the direct biochemical target of an inhibitor (i.e. to which it physically binds and inhibits), but also genes in the same pathway, depletion of which can also be sensitized by the drug. In addition, synergies can be observed with other functionally interacting genes and pathways. There are various reasons that other genes in the library might also display sensitivity to the drug when depleted, such as general stress response pathways, efflux or detoxification mechanisms, or genes that can induce shifts in the metabolic network or redox state to relieve or bypass or compensate for the drug stress [31, 32]. If multiple genes in a relevant pathway are represented in the library, it might be possible to detect the interaction through *pathway analysis*, even if the member genes are only weakly depleted individually. Even though none of the pathway members might represent outliers on their own, there might be a systematic effect where each of the pathway genes exhibits partial depletion (negative slopes that are still significantly different from 0). As a group, this could be detected as statistically significant, indicating synergy between the drug and the pathway. We observed this effect for exposure to both bedaquiline (ATP synthase genes) and copper (muramic-acid pathway). In the case of bedaquiline, pathway analysis with GSEA shows that sensitivity of detecting drug targets that are members of a complex can be enhanced because other members of the complex can collectively show depletion effects. In the case of copper, pathway analysis led to the insight that copper might be interacting, directly or indirectly, with peptidoglycan synthesis, as nearly all of the genes in the muramic acid pathway were sensitized to depletion in the presence of copper. GSEA effectively aggregates hits with similar functions that are ranked near the top by the CGA-LMM model (with most negative slopes, hence greatest fitness defect). The advantage of GSEA is that it takes the full ranking of genes into account, not just those selected as significant by a hard cutoff (such as Zrobust<-3.5).

For 7 out of 9 drugs where there is a known target gene or expected interaction in the library, the CGA-LMM analysis identified the expected target gene among the list of outliers: trimethoprim (*trpG*), methotrexate (*trpG*), levofloxacin (*gyrA*), moxifloxacin (*gyrA*), fidaxomicin (*rpoB*), sulfamethoxazole (*thyA*), and isoniazid (*ino1*, *kasB*). Expected target genes were ranked highly (with negative slopes) but not enough to be counted as outliers for rifampin (*rpoB*) and bedaquiline (ATP synthase genes). The results for all drugs evaluated are discussed in more detail below.

In the re-analysis of the 4 drugs in the dataset from [9], the CGA-LMM analysis identified 4 genes that potentially interacted with each drug. In the case of TMP and MTX, though the expected target (*dfrA*) was not present in the library, *trpG* was among the genes with outlying negative slopes (strongly depleted with increasing drug concentration), indicating synergy with both drugs. In *M*. *tuberculosis*, *trpG* is a bifunctional enzyme with glutamine aminotransferase activity to multiple substrates that feeds into both the tryptophan synthesis pathway and folate pathway [33, 34]. For the tryptophan pathway, *trpG* (as anthranilate synthase, in complex with *trpE*) synthesizes anthranilate from chorismate. For the folate pathway, *trpG* (as deoxychorismate synthase, in complex with *pabB*) synthesizes 4-amino-4-deoxychorismate (ADC), which is subsequently converted into folate, which is then utilized as a substrate by *dfrA* [35]. Hence the negative interactions observed between *trpG* depletion and the *dfrA* inhibitors (TMP and MTX) could be explained by the increased sensitivity to metabolic flux into the folate pathway at the entry point. Johnson et al. (2019) validated this interaction by making the knock-down mutant of *trpG* and showing that it is more sensitive to antifolate drugs.

For RMP, the expected target, *rpoB*, was ranked #14 out of 152 genes by the CGA-LMM analysis. While the slope was not negative enough to be labeled as an outlier, it is ranked more highly than in the analysis by ConCensusGLM, for which *rpoB* was tied with 49 out of 152 genes with a p-value of 0. The fact that *rpoB* was not the most significantly depleted gene could be due to the fact that *M*. *tuberculosis* is especially sensitive to depletion of *rpoB*, making the read-count data noisy especially at higher concentrations causing more severe growth impairment leading to lower culture densities. Supporting this, *rpoB* is ranked as the 231^st^ most vulnerable gene to depletion out of 4052 genes in the Mtb genome [11]. It is intriguing that *dapF* was also identified as a potential C-G interaction with RMP. *dapF* is in the pathway for synthesizing diaminopimelate, which ultimately gets incorporated into peptidoglycan in the cell wall. Although not a direct target of RMP, the DAP pathway has recently been shown to interact with rifampicin treatment in a separate C-G experiment (Koh et al, in review, bioRxiv pre-print, https://doi.org/10.1101/2021.04.08.439092).

The analysis of BRD-4592 in this dataset is challenging because the expected target, *trpA*, was not present in hypomorph. Although 4 genes with outlying slopes were identified for BRD-4592, they do not bear any interpretable relationship to the known mechanisms of action. It is possible that BRD-4592 might have functional interactions with other genes (in addition to *trpA*) that are not represented in the hypomorph library, since the hypomorph library contains only about one-quarter of the essential genes (152 out of 625) in the H37Rv genome [36].

In the analysis of the data we collected from a hypomorph library with 162 essential genes, we observed that *gyrA* gave a strong signal of depletion and was among the top 2 interactions for both fluoroquinolones, levofloxacin and moxifloxacin. Although there were several other genes with outlier negative slopes, such as *asnB*, their relevance to fluoroquinolone resistance remains to be investigated. Interestingly, a transposon-insertion mutant of *asnB* in *M*. *smegmatis* was found to exhibit increased sensitivity to several drugs, though not to norfloxacin [37].

For INH, the expected target, *inhA*, was not in the library. However, we observed *kasB* in the fatty-acid synthesis pathway that had a negative interaction, which is consistent with the mechanism of action of INH. Both *kasB* and *inhA* are in FAS II cycle, which is utilized for generating long-chain lipids (by extending the length of short-chain lipids produced by fatty-acid synthase) for incorporation into mycolic acids. It is plausible that other genes in the fatty-acid pathway besides the target *inhA* would become sensitized by exposure to an inhibitor of mycolic acid synthesis and hence show excessive depletion of knock-down mutants in the presence of INH. Interestingly, we also observed *ino1* to have the most outlying slope (ranked #1), which is in the mycothiol synthesis pathway. The Ino1 enzyme (inositol-3-phosphate synthase) catalyzes generates the inositol precursor as an early step in mycothiol synthesis. Mycothiol plays an important role in redox homeostasis, and INH activity has been shown to be sensitive to mycothiol levels and intracellular redox state [38].

For sulfamethoxazole (SMX), the expected target, DHPS (*folP1*), which is in the folate pathway, was not represented as a mutant in the hypomorph library. However, *thyA* (thymidylate synthase), utilizes folate as a co-factor, was observed as an outlier (rank #7). *thyA* is required for nucleotide synthesis, and it could become more sensitive to depletion when folate production is reduced, explaining the synergy (negative interaction) between SMX exposure and *thyA* depletion. We also observed that *efpA* had the second highest (positive) slope for SMX (Zrobust = +9.5). This implies that the *efpA* knock-down mutant increased in abundance with increasing drug concentration. This effect was unique for sulfamethoxazole. *efpA* is an essential efflux pump in the cell membrane [9]. Although the substrate(s) of *efpA* are unknown, this selection for the *efpA* knock-down mutant by sulfamethoxazole would be consistent with either *efpA* facilitating uptake of SMX, or pumping a metabolite out of the cell whose synthesis is interfered with by SMX, resulting in tolerance.

The treatment with BDQ led to interactions with ATP synthase genes (4 of the 8 subunits were in the hypomorph library: *atpB*, *atpF*, *atpG*, *atpH*). Although none of them had an outlier slope on their own, they all showed a consistent negative trend with increasing drug concentration, and pathway analysis with GSEA showed that this was statistically significant. This shows that multiple members of a complex (like the ATP synthase) can exhibit a sensitivity to a drug (like BDQ) similar to the subunit to which it directly binds (*atpE*), thus enhancing the detection of a C-G interaction signal.

We also used CGA-LMM to analyze data from a hypomorph library to determine chemical-genetic interactions with copper. Copper is known to be bactericidal at high concentrations for many bacteria, and copper toxicity is relevant to pathogenesis for *M*. *tuberculosis*, as macrophages have been shown to secrete copper into phagosomes as one of several defense mechanisms to destroy infecting TB bacilli [39, 40]. Excess copper can cause a variety of problems in cells, including general oxidative damage, as well as displacement of cognate metal ions in metal-binding proteins [30, 41]. Although the precise mechanism of copper toxicity in Mtb is not known, studies in other organisms have implicated interference with cell-wall maintenance as a potential mechanism of action [28, 29]. To evaluate genes and pathways interacting with copper in *M*. *tuberculosis*, we treated an Mtb hypomorph library with copper and used CGA-LMM to look for synergistic behavior. The library was grown on three different carbon sources–glycerol, acetate, and cholesterol–in recognition of the distinct changes in metabolism induced by each [42], with the latter 2 representing lipid sources thought to be utilized in vivo [43–45]. The top interacting gene with copper observed in all 3 carbon sources was TrxB2, which is a thioredoxin reductase. It is plausible that knock-down mutants of TrxB2 might be more sensitive to copper exposure, since high copper concentrations increase intracellular redox potential (through generation of oxygen radicals, etc) [46], and thioredoxin reductases help maintain redox homeostasis [20] and could thus help mitigate some of the consequential oxidative damage [30]. However, this interaction with TrxB2 has yet to be validated experimentally.

Pathway analysis of the CGA-LMM results for copper pointed to an enrichment of genes in the peptidoglycan pathway, which was the most enriched functional category. In particular, 6 of the 7 genes in the hypomorph library associated with this category were in the muramic-acid synthesis pathway–*murACDEFX*–which all exhibited sensitivity (excess depletion) with increasing copper concentrations. Muramic acid is a constituent of lipid I, to which cytoplasmically-assembled pentapeptides are attached and transported across the lipid membrane to the mycobacterial periplasmic space and incorporated into peptidoglycan. Studies in other organisms have also found that copper interferes with peptidoglycan synthesis. In *E*. *coli*, knock-outs of Ldt genes, L,D-transpeptidases that cross-link peptidoglycan, were shown to be more sensitive to copper, as well as affecting other measures of cell-wall integrity, and *E*. *coli* strains that specifically rely on Ldt’s for β-lactam resistance were shown to have increased β-lactam sensitivity in the presence of CuCl_2_ [28]. Similarly, the interaction we observed (i.e. synergy between depletion of *mur* genes and increasing copper concentration) suggests that copper either interferes directly with muramic-acid synthesis, or it could be indirectly interfering more broadly with peptidoglycan synthesis, resulting in increased sensitivity to synthesis of this critical component on which it relies (muramic acid, in lipid I). Unfortunately, none of the 5 Ldt genes in the Mtb genome was represented in the hypomorph library. The library also did not contain any of the genes of the *ricR* or *csoR* operons (e.g. *ctpV*, *mymT*, *mmcO*…), which have been shown to provide tolerance of copper at moderate levels in mycobacteria [47], and which we might have predicted would have also exhibited negative interactions. While the *mur* genes did not have as extreme of a negative slope as TrxB2 (and only 3 of 6 *mur* genes exceeded the cutoff for outliers), they all showed a negative trend of abundance with increasing copper concentration (slope) that was significant, and the high ranking (by Zrobust) of these 6 genes as a group was statistically significant by pathway analysis (GSEA). Interestingly, this enrichment appeared to be specific to growth on cholesterol as a carbon source, as the enrichment of the *mur* pathway was not significant when the library was grown on acetate or glycerol and treated with copper. It is possible that this is a consequence of metabolic changes that are known to occur when cholesterol is catabolized (especially odd-chain-length lipids) [21]. While the mechanism underlying this observation is not clear, it is notable that both cholesterol catabolism and copper toxicity are relevant during infection, making this interaction a potentially important determinant of pathogenesis.

Sometimes relevant genes that were expected to interact with a drug failed to make the cutoff (Zrobust<-3.5) and were thus not identified as outliers, as in the case of *rpoB* for rifampin, and the ATP synthase genes for bedaquiline (even though were generally ranked highly, and had negative slopes that were significantly different than zero, indicating depletion). There are several reasons why an expected target gene might not exhibit an outlier negative interaction. First, the concentrations evaluated in the experiment might not span the best range to observe the drop-off in abundance (discussed further below). Second, the strength of depletion of a target protein might not be optimized. In a ClpXP knock-down library, the amount of depletion depends on the level of expression of the *sspB* protein (which depends on the promoter strength used), and some proteins are more sensitive to depletion than others [4, 11]. A strong signal for synergy depends on both of these parameters together–drug concentration range and level of protein depletion. There might be additional experimental parameters that also influence the effectiveness of detecting synergy, such as the amount of time allowed for pre-depletion of proteins levels (which is a function of protein stability and degradation rate). Thus, it seems prudent to use Zrobust<-3.5 as a guide but not a strict cutoff for identifying interactions. Still, the ranking of genes by slope (i.e. concentration-dependent depletion of mutant abundance) provides as meaningful way to prioritize genes in a hypomorph library for follow-up studies. We also note that some genes appear on the list of outliers for multiple drugs. Some of these might represent target-independent mechanisms of drug-tolerance [48]. An example of this might be *asnB* (asparagine synthetase), which appears on the list of candidate interactions for four drugs in 3 different classes in Table 3 (levofloxacin, moxifloxacin, isoniazid, and fidaxomycin), as it has been implicated in resistance to multiple drugs [37].

The ideal number of concentrations to use in a chemical-genetics experiment is an important logistical question. Typically, concentrations are chosen in a range just below the MIC of the compound, since concentrations above the MIC inhibit growth of the entire culture. Concentrations just below the MIC are needed so that there is some biological pressure applied by the drug, to enable an opportunity to observe the synergy with protein depletion mentioned above. (Above the MIC, the culture will likely experience too much growth impairment, resulting in low OD (optical density), making read-out of DNA barcodes noisy or infeasible) On the one hand, the more concentrations evaluated, the better, allowing better fits in the regression model, and a survey of a greater range of concentrations to increase the probability of identifying concentration-depending depletion effects. On the other hand, evaluating more concentrations increases the cost of the experiment drastically. While two concentrations would be the minimum for fitting a line, it is probably advisable to use 3–5 concentrations, in order to better detect trends. In fact, over a wider range of concentrations (say 8 to 10 2-fold dilutions), there might exist a "sweet spot" for observing the interaction between a drug and target gene, where there is little depletion of mutant abundance at lowest concentrations, then a transition, converging to full depletion at higher concentrations, although the exact concentration at which this transition occurs might be difficult to predict ahead of time. While a regression-based approach could still capture such trends, the sensitivity of the detection might be increased by applying the LMM over just sub-ranges of concentrations, for example in a sliding window of 3–5 concentrations at a time, which might be able to detect the interaction with higher significance when focused on the range containing the transition. A sensitivity analysis for detection of target genes for trimethoprim and rifampin is provided in S1 File, which shows that, while similar results are obtained even when using different subsets of concentrations in the regression analysis (though doses above the MIC are not as informative for the model as lower doses), it likely going to be difficult to anticipate in an agnostic way the optimal concentration range where synergy will be observed for a given drug-gene interaction.

In summary, the CGA-LMM method can be used to analyze data from hypomorph libraries (sequencing barcode counts that represent abundance of each knock-down mutant), and takes advantage of dose-dependent depletion to look for genes that synergize with a drug to improve the identification of genuine chemical-genetic interactions. A linear-mixed model (LMM) is used to quantify the concentration dependence of gene mutant abundance as random-effect coefficients (slopes), and we showed that employing outlier analysis of distribution of slopes produces a shorter, more focused list of candidate genes, among which we found known interactions for multiple antibiotics.

## Supporting information

S1 File Plots for all drugs investigated. Sensitivity analyses.
(PDF)

S1 Table Data_Summary–statistics on datasets for each drug. (XLSX)

S2 Table Results of CGA-LMM analysis for trimethoprim, methotrexate, rifampin, BRD-4592 in first hypomorph library. (XLSX)

S3 Table Barcode counts for treatment of second *M*. *tuberculosis* hypomorph library with levofloxacin, moxifloxacin, isoniazid, fidaxomicin, sulfamethoxazole, and bedaquiline. (XLSX)

S4 Table Results of CGA-LMM analysis for levofloxacin, moxifloxacin, isoniazid, fidaxomicin, sulfamethoxazole, and bedaquiline in second hypomorph library. (XLSX)

S5 Table Barcode counts for copper treatment. (XLSX)

S6 Table Results of CGA-LMM analysis of interactions with copper in third hypomorph library. (XLSX)
