# Supplementary material for: An improved statistical method to identify chemical-genetic interactions by exploiting concentration-dependence
Source: PLoS One. 2021 Oct 1;16(10):e0257911. doi: 10.1371/journal.pone.0257911 (PMC8486102; doi:10.1371/journal.pone.0257911)
Supplement: S1 File — Sensitivity analyses. (PDF) [file pone.0257911.s001.pdf]

An Improved Statistical Method to Identify Chemical-Genetic Interactions by Exploiting Concentration-Dependence

Esha Dutta, Michael A. DeJesus, Nadine Ruecker, Anisha Zaveri, Eun-Ik Koh, Christopher M. Sassetti, Dirk Schnappinger, and Thomas R. Ioerger

SUPPLEMENTAL INFORMATION

1. Abundance plots and slope histograms for all drugs

Trimethoprim

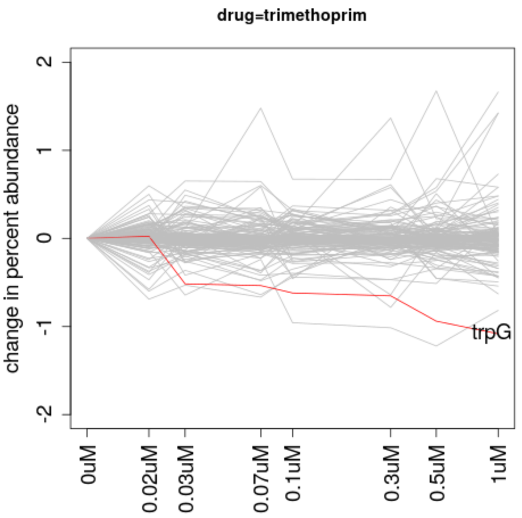

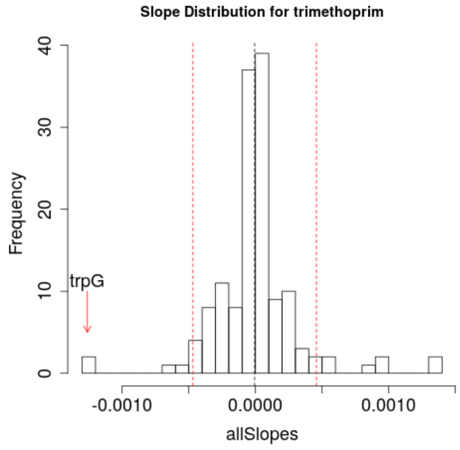

Methotrexate

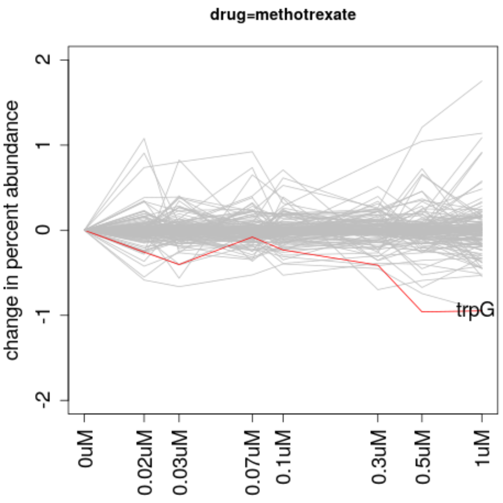

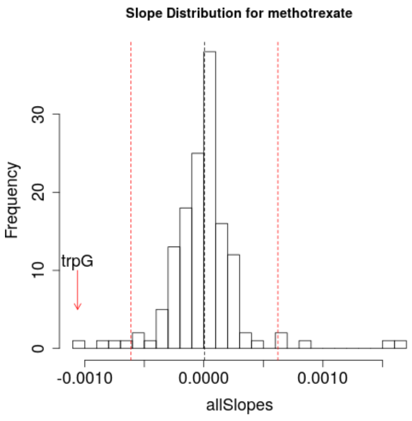

Rifampin

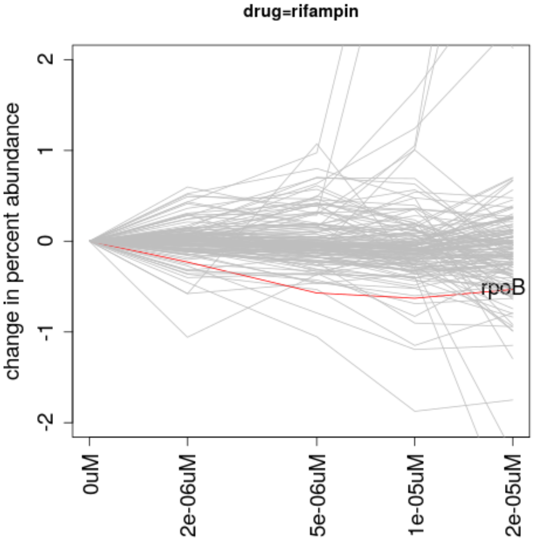

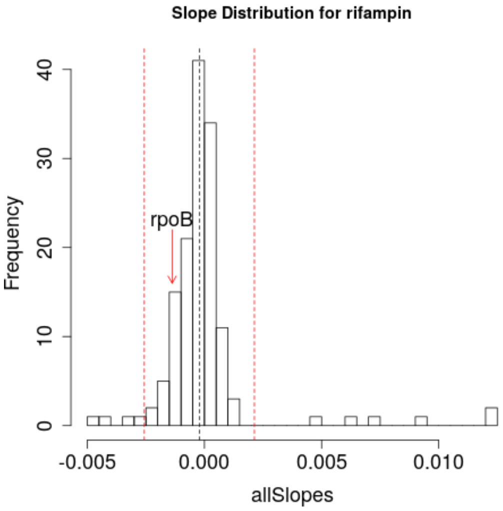

BRD-4592

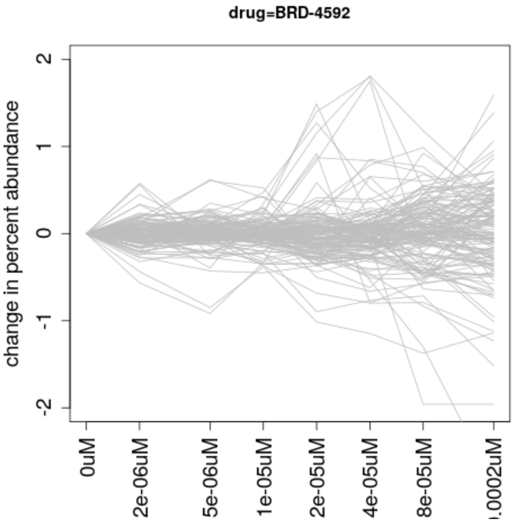

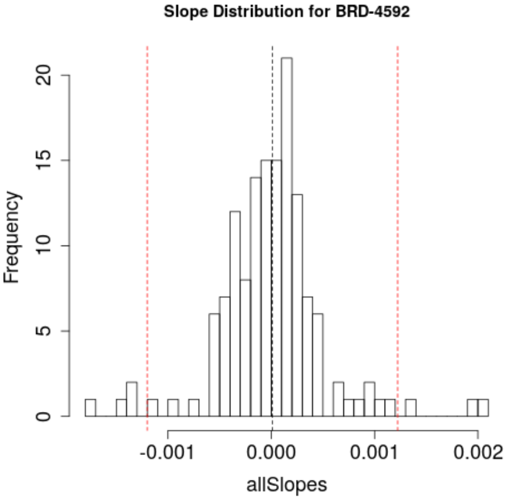

Levofloxacin

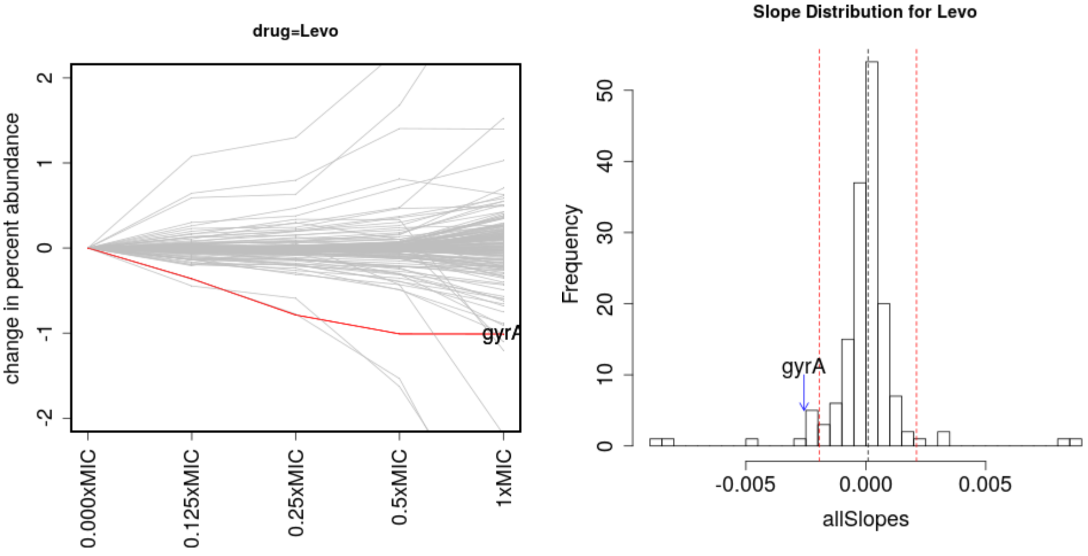

Moxifloxacin

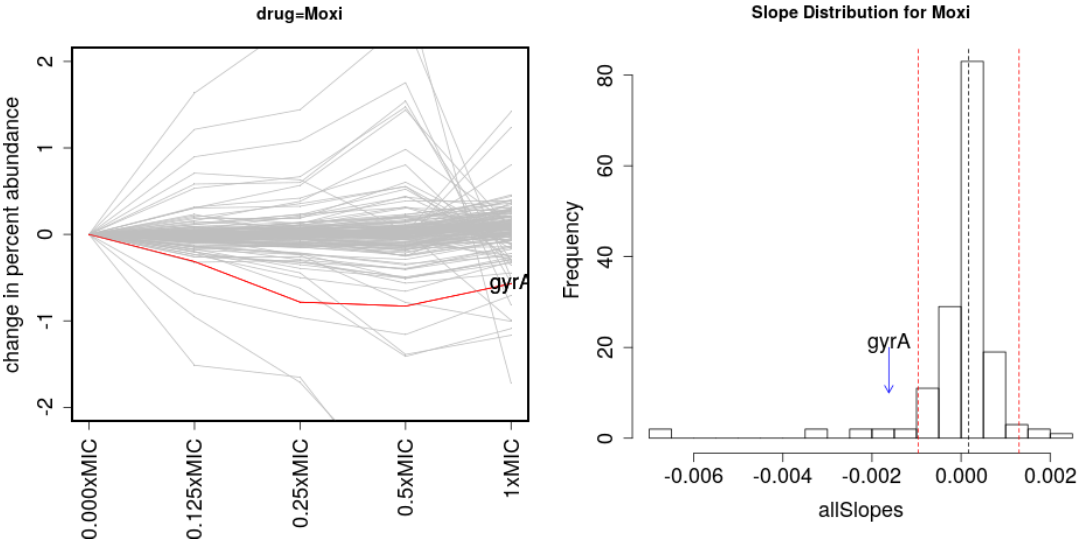

Isoniazid

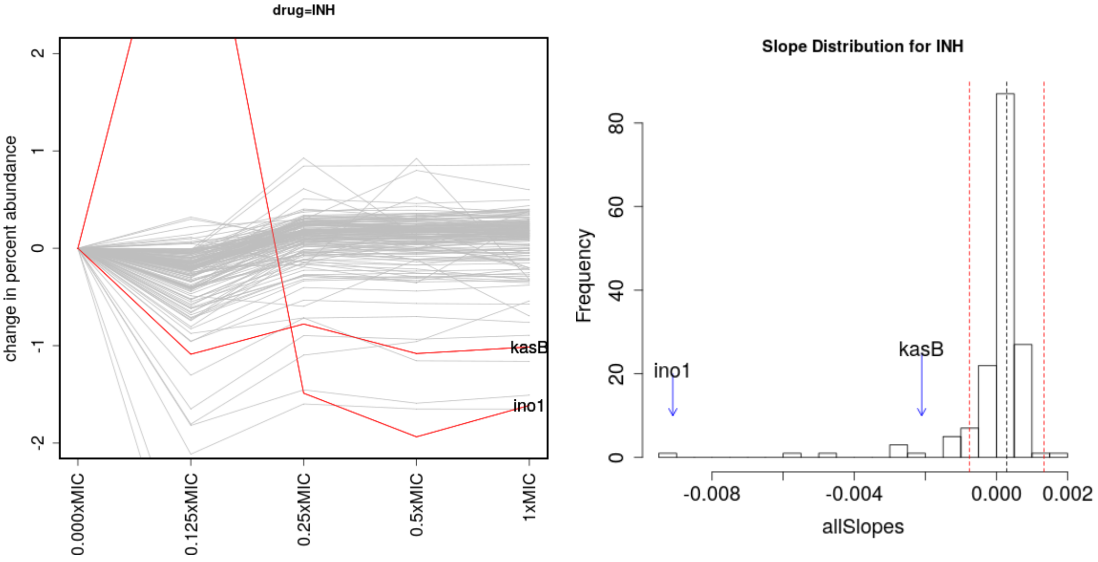

Fidaxomycin

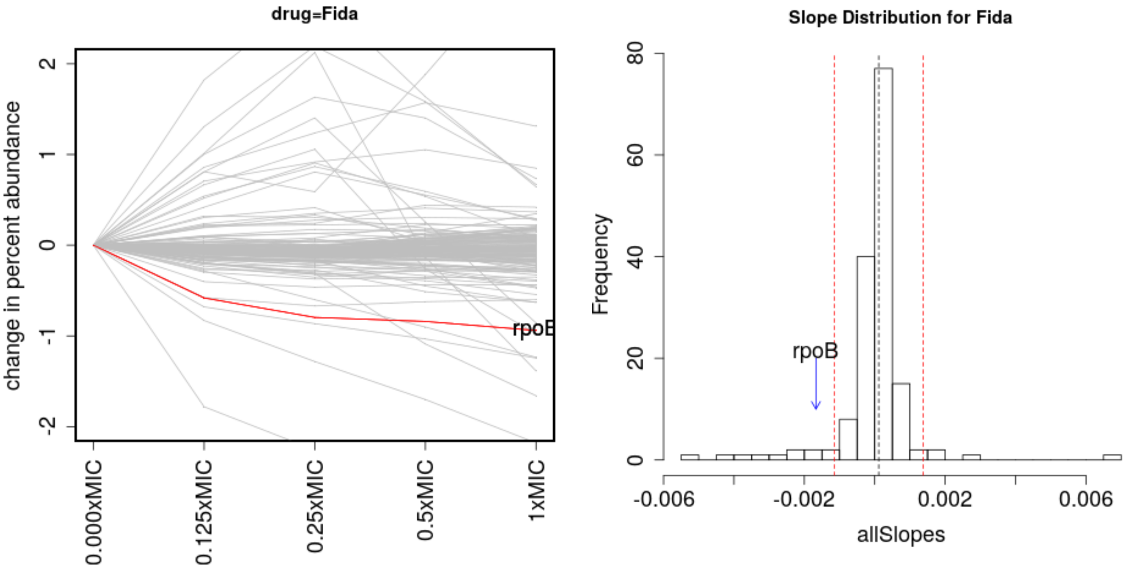

Sulfamethoxazole

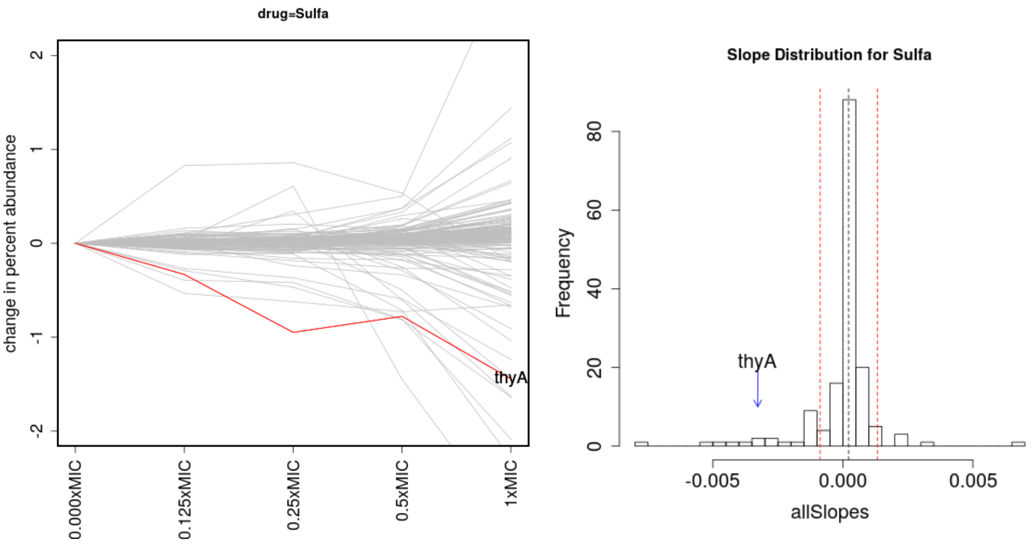

Bedaquiline

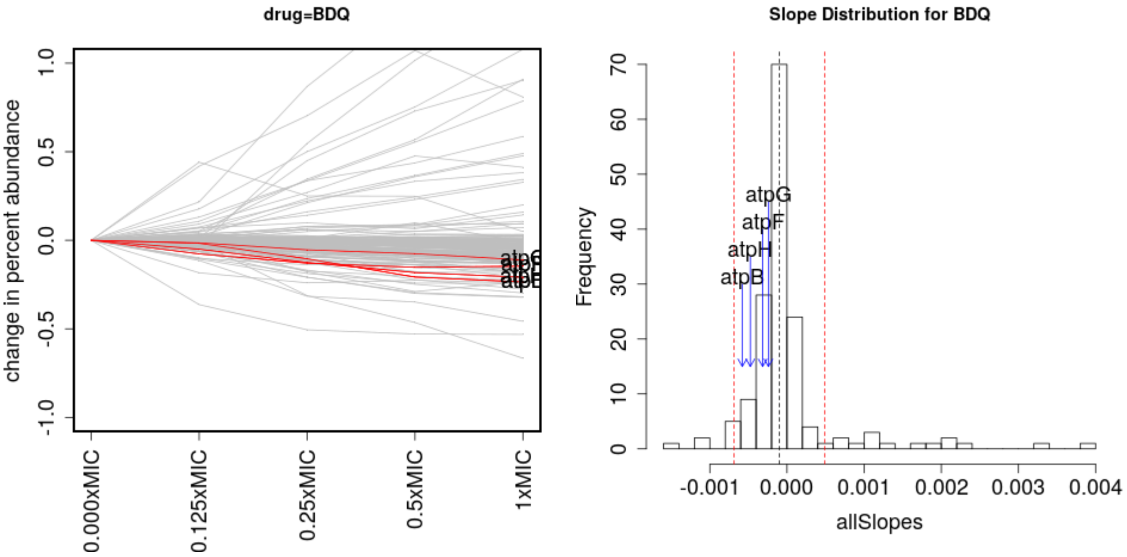

Cu (grown on cholesterol as a carbon source)

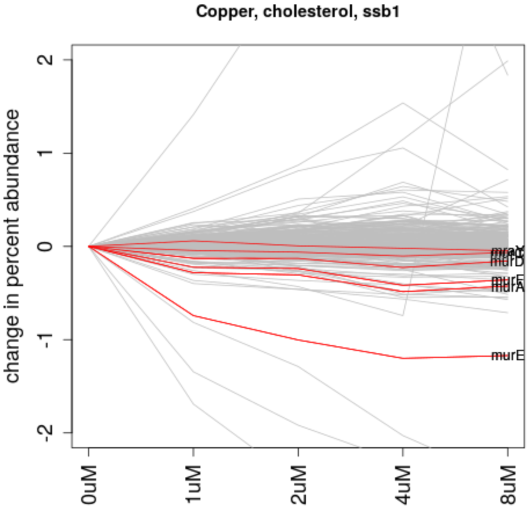

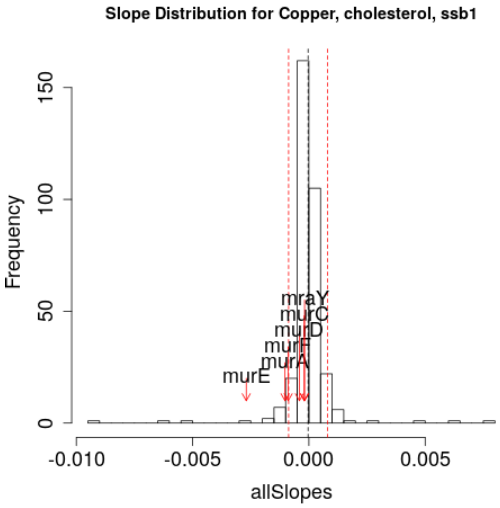

Cu (grown on acetate as a carbon source)

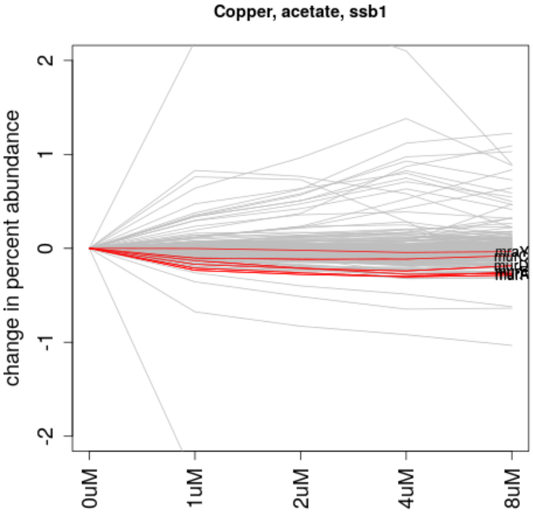

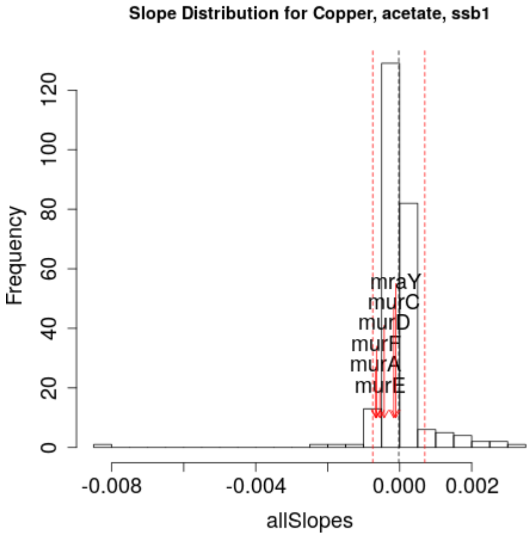

Cu (grown on glycerol as a carbon source)

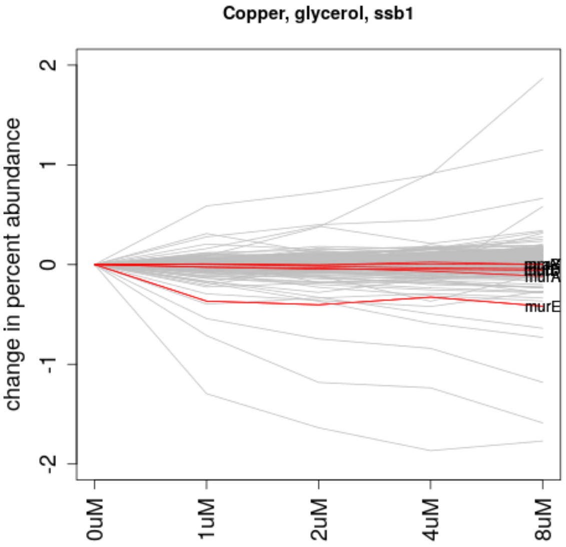

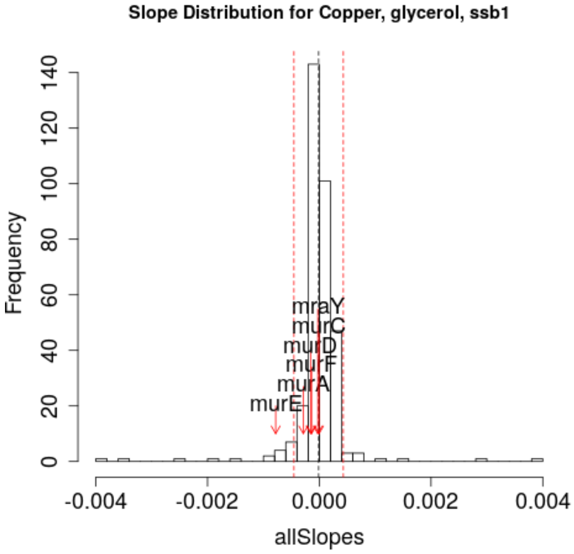

2. Sensitivity Analysis

To determine the impact of number and range of concentrations used in the regression on the ability to identify chemical-genetic interactions, we performed an analysis where the LMM was fit to different subranges of concentrations and examined the Zrobust score for two drugs where an expected target is known: *trpG* for trimethoprim (TMP), and *rpoB* for rifampin (RMP). The objective of this analysis is to explore how using different concentration ranges would affect to the sensitivity to detect the interactions with these genes. We chose to use Zrobust instead of slope of the target gene as the metric because the distribution of slopes over all genes could be different depending on which subset of data the model is fitted to; Zrobust factors that variability into an independent measure of significance for the interacting gene that can be compared between the different models.

The left plot below shows the data for TMP over the full range of concentrations (from the Broad dataset), with the relevant interacting gene *trpG* highlighted. On the right, the Zrobust score for *trpG* is shown for LMMs fit on different subranges of concentrations. For example, the reddest cell (with Zrobust=-9.3) is for concentrations starting at 0uM and going to 0.5uM (7 consecutive concentration points). However, there are multiple subranges which also have a Zrobust score of below -3.5 for *trpG*, showing that it would be detected as an interaction regardless of which subrange of concentrations was used. The exceptions are for concentrations ranges that go above ~1uM (final conc, upper end of subrange). This means that the outlier negative slope is evident at lower concentrations, but as abundance data for higher concentrations is included, it decreases the magnitude of the slope for *trpG*, until it is indistinguishable from the rest of the population. This is also evident in the plot on the left; *trpG* abundance decreases strongly until around 1uM, and then starts increasing, which will make the slope from the regression less negative. Interestingly, there is one subrange spanning only 3 concentrations (0uM-0.03uM) where *trpG* is significant (Zrobust=-5.1). However, for most other ranges spanning just 3 concentrations, the interaction is not detected. The green heatmap shows that the most outliers are detected by concentrations subranges starting at either 0uM or 0.3uM and including only 3-5 concentrations in the regression (i.e. smaller than the full range). This means it is preferable to do the CGA-LMM analysis with lower concentrations for trimethoprim, and could exclude some of the higher-concentration data points.

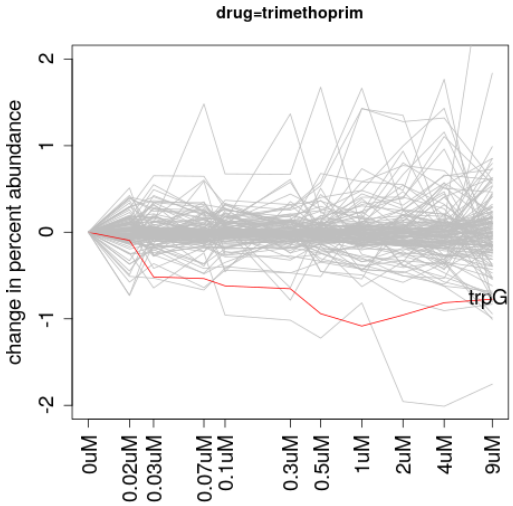
drug=trimethoprim
change in percent abundance
trpG
2
1
0
-1
-2
0uM
0.02uM
0.03uM
0.07uM
0.1uM
0.3uM
0.5uM
1uM
2uM
4uM
9uM

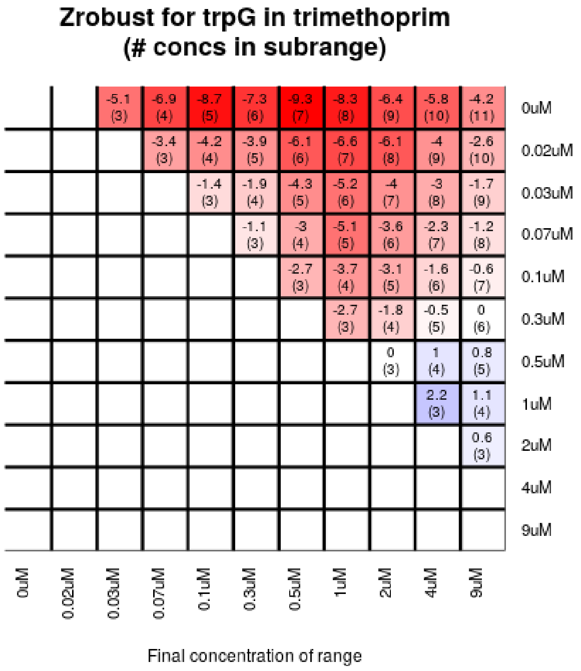
Zrobust for trpG in trimethoprim
(# concs in subrange)
Final concentration of range
0uM
0.02uM
0.03uM
0.07uM
0.1uM
0.3uM
0.5uM
1uM
2uM
4uM
9uM

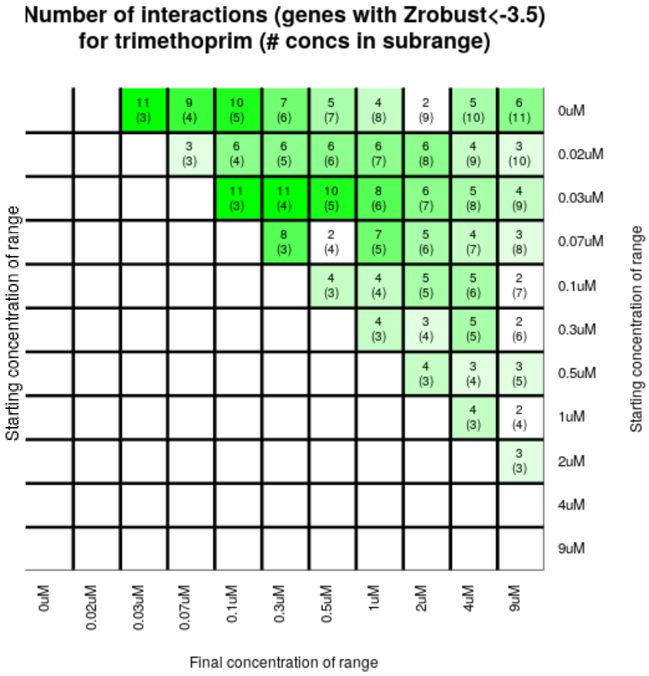
Number of interactions (genes with Zrobust<-3.5)
for trimethoprim (# concs in subrange)
Starting concentration of range
Final concentration of range
0uM
0.02uM
0.03uM
0.07uM
0.1uM
0.3uM
0.5uM
1uM
2uM
4uM
9uM

We repeated the same sensitivity analysis for rifampin, looking at how fitting the LMM with data from different subranges of concentration affects the significance (Zrobust) of the slope for *rpoB*. In this case, the depletion of the *rpoB* mutant is most evident at low concentrations, and is most significant for the first 3 concentrations (0uM..5e-6uM), though even here, it is not an outlier (Zrobust=-3.3). At higher concentrations, the abundance of *rpoB* creeps back up, making the slope less negative (and hence still not an outlier). For this drug, more (and higher) concentrations is clearly worse for detecting the interaction with *rpoB*. The largest number of outliers (9) is found by using only the first 3 concentrations. If the concentration range starts around 1e-5 uM, there are a few more hits (rows 4 and 5 in the green heatmap), but this excludes the lower concentrations of RMP where the slope for *rpoB* is negative.

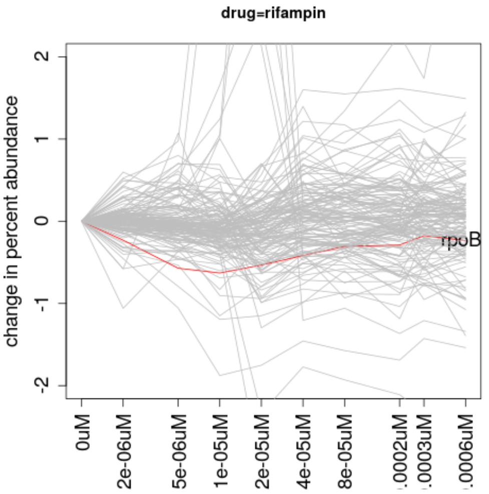

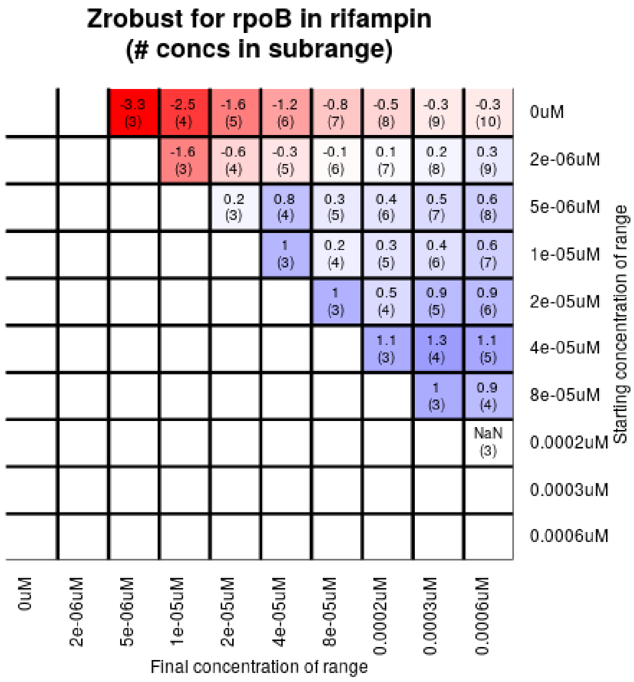

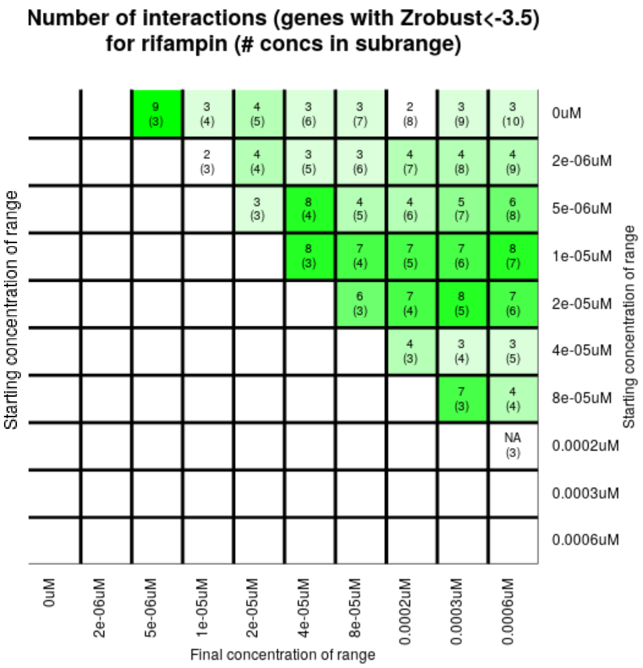

In fact, we only fit the LMM with data from the first 5 concentrations for RMP (up to 2e-5uM) because samples for higher concentrations got automatically filtered out because the total barcode counts were insufficient for those samples. This was probably due to the fact that growth was severely impaired at these higher drug concentrations, leading to a low OD600 in those wells. The lower yield of DNA from such wells can be expected to cause higher variability in the gene abundances for samples with low barcode counts. The correlation plots between concentrations supports this, showing a significant divergence in correlation between concentrations below 1uM vs above 1uM for RMP. For trimethoprim, the gene abundances only began to diverge at the highest concentration point (9uM).

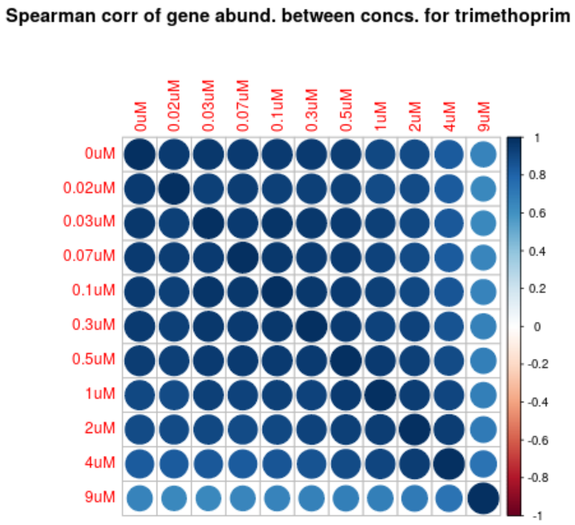

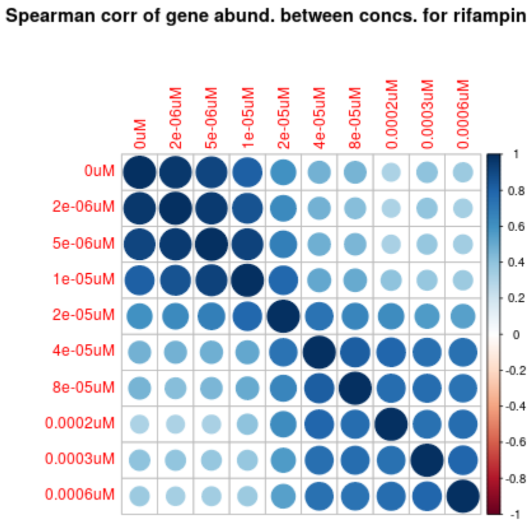

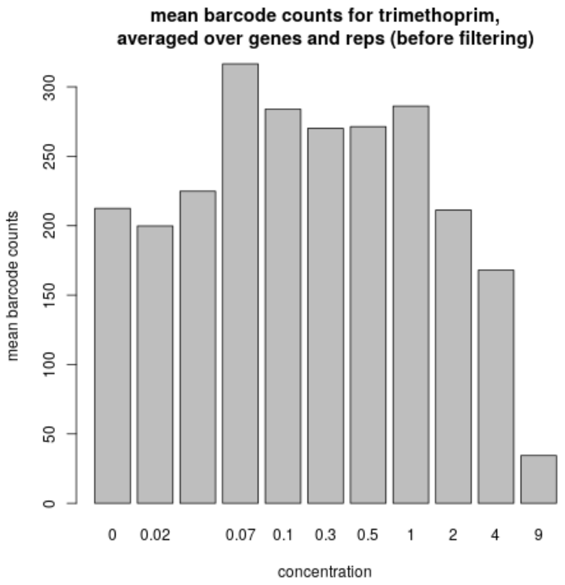

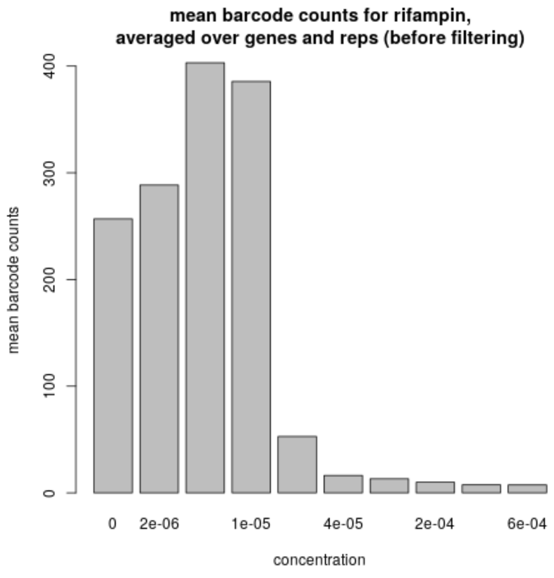

To determine the impact of number of concentrations used in the regression on the ability to identify chemical-genetic interactions, we fit the LMM for different subsets of concentrations on the trimethoprim data (TMP). In the TMP data, there were 8 concentrations spanning 0uM to 1uM (see first plot below). We chose random subsets of *k* concentrations between 0uM and 1uM, always including the 2 endpoints (so for example, in the case of *k*=3, we chose 0uM, 1uM, and one random concentration in between). Then we fit the LMM and calculated both the number of candidate interactions (with Zrobust<-3.5) and the Zrobust score for the known interaction, *trpG*. We chose this approach because it is better than comparing the slope estimate itself between models based on different concentration data points, since the slopes of all the other genes might be affected too, and Zrobust converts the slope into a significance which can be compared more fairly between models trained with different numbers of concentrations. The error bars in the plots show the range over 5 random samples of *k* concentrations. As can be seen, the number of hits (outliers with negative slopes) and the significance of *trpG* are relatively insensitive to the number of concentrations used to fit the model. For each run, there were 4-6 genes with outlier negative slopes, and the Zrobust score for *trpG* was fairly stable at around -7 to -9 .

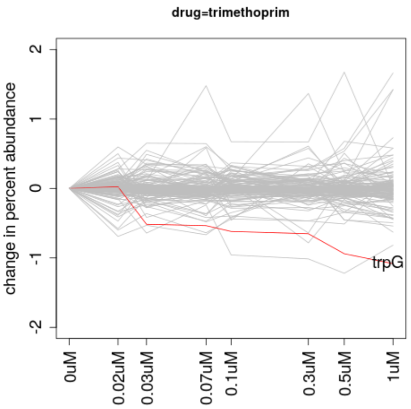

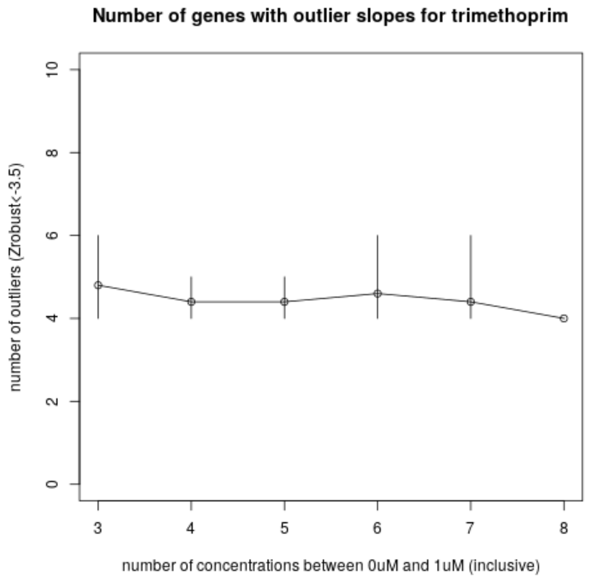

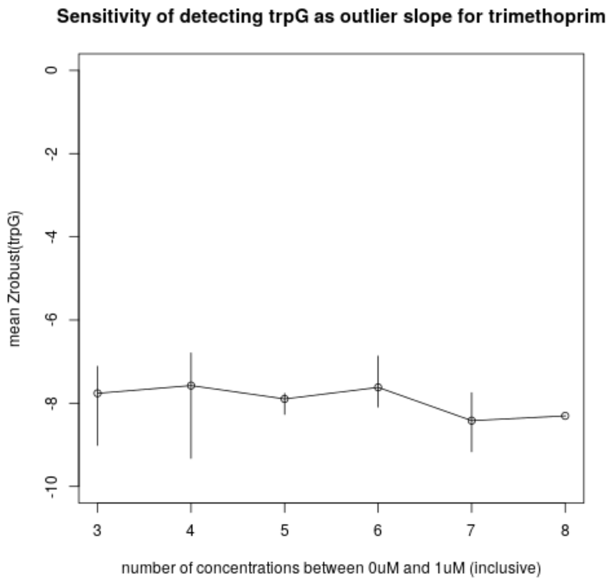

So we draw 3 conclusions from this Sensitivity Analysis, using these two example drug-gene pairs. First, whether a given interaction is detected is not totally dependent on the conc range. For TMP, the Zrobust score for *trpG* was below -3.5 for multiple ranges of concentrations below ~1uM. On the other hand, the interaction between *rpoB* and RMP was not detectable (as an outlier) for any subrange. Second, it is likely that there exists an optimal subrange of concentrations that will maximize the detection of the significance of a given interaction, and that additional concentrations only makes it look like less of an outlier. But this can only be known post-hoc. Third, the optimal concentration range to use (that spans a concentration where the synergy between a drug and depletion of a gene is most evident) is hard to anticipate a priori; it likely differs from drug to drug, and from gene to gene. It would be very difficult to provide a rigorous prescription for defining the optimal concentration range to be used in C-G experiments to look for interactions with novel inhibitors whose MOA is unknown in an agnostic way.

Effect of Treatment of No-Drug Concentration

In the CGA-LMM method as described, we treat the no-drug control as a concentration 2-fold lower than the lowest concentration of drug measured in the experiment (because the concentrations are log-transformed prior to doing the regressions and we cannot take log of 0, and most of these experiments are done with 2-fold dilutions anyway). In order to assess the impact of different choices for including the no-drug control, we re-ran the analysis on the levofloxacin data with alternative choices for the no-drug concentration – 4x, 8x, and 16x lower than the lowest drug concentration – and evaluated the effect on the number of outliers, and the rank and significance of the target gene, *gyrA*. The results in the Table below show that, although the slope of the regression for *gyrA* in the LMM flattens-out (becomes less negative) as the no-drug concentration decreases, the number of outliers stays relatively constant at 9 (except for the most extreme case), *gyrA* is always ranked as the 4th-most depleted gene (in terms of mutant abundance), and the Zrobust score for *gyrA* actually increases slightly. This is because, although lowering the no-drug concentration flattens-out the regression line for *gyrA*, it also flattens-out the slopes of the rest of the population, so the relative significance, as quantified by Zrobust, stays fairly stable. The conclusion we draw is that the CGA-LMM method in detecting C-G interactions is relatively insensitive to the choice of concentration (on a log scale) used for including the no-drug control in the regressions.

Table. Results of treating the no-drug control as different values in the LMM for levofloxacin.

no-drug Conc	slope for *gyrA*	Zrobust for *gyrA*	# genes with outlier neg. slopes	rank for *gyrA*
1/16xMIC*	-0.00257	-4.615	9	#4
1/32xMIC	-0.00217	-4.919	9	#4
1/64xMIC	-0.0018	-5.194	9	#4
1/128xMIC	-0.00152	-5.528	7	#4

* 2x lower than the lowest concentration of levofloxacin used (1/8=0.125xMIC)

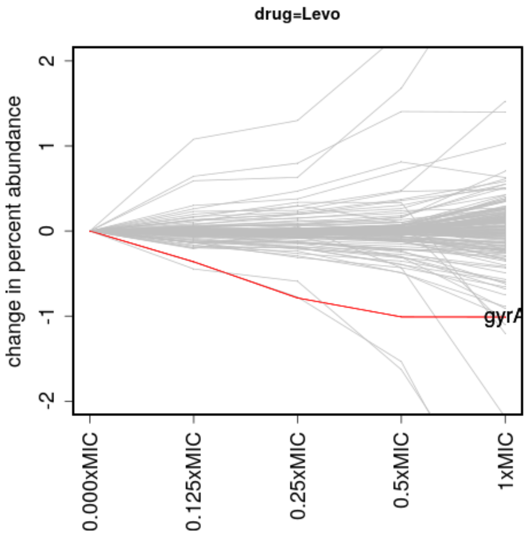

no-drug treated as 1/16xMIC

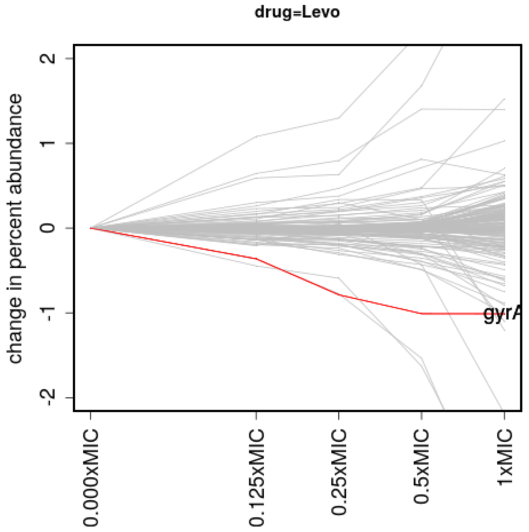

no-drug treated as 1/32xMIC

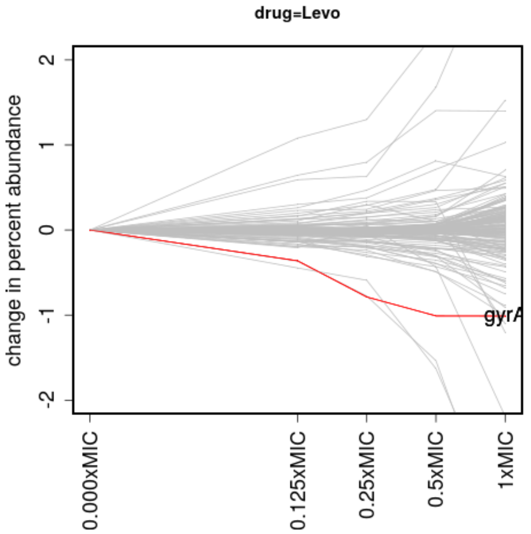

no-drug treated as 1/64xMIC

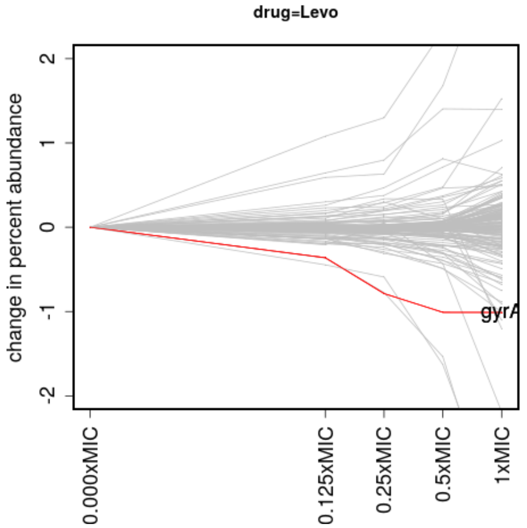

no-drug treated as 1/128xMIC
